# Genome-wide association analyses identify 143 risk variants and putative regulatory mechanisms for type 2 diabetes

**DOI:** 10.1038/s41467-018-04951-w

**Published:** 2018-07-27

**Authors:** Angli Xue, Yang Wu, Zhihong Zhu, Futao Zhang, Kathryn E. Kemper, Zhili Zheng, Loic Yengo, Luke R. Lloyd-Jones, Julia Sidorenko, Yeda Wu, eQTLGen Consortium, Mawussé Agbessi, Habibul Ahsan, Isabel Alves, Anand Andiappan, Philip Awadalla, Alexis Battle, Frank Beutner, Marc Jan Bonder, Dorret Boomsma, Mark Christiansen, Annique Claringbould, Patrick Deelen, Tõnu Esko, Marie-Julie Favé, Lude Franke, Timothy Frayling, Sina Gharib, Gregory Gibson, Gibran Hemani, Rick Jansen, Mika Kähönen, Anette Kalnapenkis, Silva Kasela, Johannes Kettunen, Yungil Kim, Holger Kirsten, Peter Kovacs, Knut Krohn, Jaanika Kronberg-Guzman, Viktorija Kukushkina, Zoltan Kutalik, Bernett Lee, Terho Lehtimäki, Markus Loeffler, Urko M. Marigorta, Andres Metspalu, Lili Milani, Martina Müller-Nurasyid, Matthias Nauck, Michel Nivard, Brenda Penninx, Markus Perola, Natalia Pervjakova, Brandon Pierce, Joseph Powell, Holger Prokisch, Bruce Psaty, Olli Raitakari, Susan Ring, Samuli Ripatti, Olaf Rotzschke, Sina Ruëger, Ashis Saha, Markus Scholz, Katharina Schramm, Ilkka Seppälä, Michael Stumvoll, Patrick Sullivan, Alexander Teumer, Joachim Thiery, Lin Tong, Anke Tönjes, Jenny van Dongen, Joyce van Meurs, Joost Verlouw, Uwe Völker, Urmo Võsa, Hanieh Yaghootkar, Biao Zeng, Allan F. McRae, Peter M. Visscher, Jian Zeng, Jian Yang

**Affiliations:** 1 Institute for Molecular Bioscience, The University of Queensland, Brisbane, Queensland 4072 Australia; 2 The Eye Hospital, School of Ophthalmology & Optometry, Wenzhou Medical University, Wenzhou, Zhejiang 325027 China; 3 Estonian Genome Center, Institute of Genomics, University of Tartu, Tartu, 51010 Estonia; 4 Queensland Brain Institute, The University of Queensland, Brisbane, Queensland 4072 Australia; 5 Computational Biology, Ontario Institute for Cancer Research, Toronto, Ontario M5G 0A3 Canada; 6 Department of Public Health Sciences, University of Chicago, Chicago, IL 60637 USA; 7 Singapore Immunology Network, Agency for Science, Technology and Research, Singapore, 138648 Singapore; 8 Department of Computer Science, Johns Hopkins University, Baltimore, MD 21218 USA; 9 Heart Center Leipzig, Universität Leipzig, 04289 Leipzig, Germany; 10 Department of Genetics, University Medical Centre Groningen, 9713 GZ Groningen, The Netherlands; 11 Faculty of Genes, Behavior and Health, Vrije Universiteit Amsterdam, 1081 HV Amsterdam, The Netherlands; 12 Cardiovascular Health Research Unit, University of Washington, Seattle, WA 98195 United States of America; 13 Estonian Genome Center, University of Tartu, 50090 Tartu, Estonia; 14 Exeter Medical School, University of Exeter, Exeter, EX4 4QD UK; 15 Department of Medicine, University of Washington, Seattle, WA 98195 USA; 16 School of Biological Sciences, Georgia Tech, Atlanta, GA 30332 USA; 17 MRC Integrative Epidemiology Unit, University of Bristol, Bristol, BS8 1TH UK; 18 Department of Clinical Physiology, Tampere University Hospital, 33521 Tampere, Finland; 19 Faculty of Medicine and Life Sciences, University of Tampere, 33100 Tampere, Finland; 20 National Institute for Health and Welfare, University of Helsinki, 00100 Helsinki, Finland; 21 Institut für Medizinische InformatiK, Statistik und Epidemiologie, LIFE – Leipzig Research Center for Civilization Diseases, Universität Leipzig, 04103 Leipzig, Germany; 22 IFB Adiposity Diseases, Department of Medicine, Universität Leipzig, 04103 Leipzig, Germany; 23 Interdisciplinary Center for Clinical Research, Faculty of Medicine, Universität Leipzig, 04103 Leipzig, Germany; 24 Lausanne University Hospital, 1011 Lausanne, Switzerland; 25 Department of Clinical Chemistry, Fimlab Laboratories and Faculty of Medicine and Life Sciences, University of Tampere, 33110 Tampere, Finland; 26 Institute of Genetic Epidemiology, Helmholtz Zentrum München, 81377 München, Germany; 27 Institute of Clinical Chemistry and Laboratory Medicine, University Medicine Greifswald, 17489 Greifswald, Germany; 28 Institute of Human Genetics, Helmholtz Zentrum München, 81675 München, Germany; 29 Cardiovascular Health Research Unit, Departments of Epidemiology, Medicine, and Health Services, University of Washington, Seattle, WA 98195 USA; 30 Department of Clinical Physiology and Nuclear Medicine, Turku University Hospital, 20521 Turku, Finland; 31 University of Turku, 20500 Turku, Finland; 32 School of Social and Community Medicine, University of Bristol, Bristol, BS8 1TH UK; 33 Department of Medical Epidemiology and Biostatistics, Karolinska Institute, 171 77 Solna, Sweden; 34 Institute for Community Medicine, University Medicine Greifswald, 17489 Greifswald, Germany; 35 Institute for Laboratory Medicine, LIFE – Leipzig Research Center for Civilization Diseases, Universität Leipzig, 04107 Leipzig, Germany; 36 Division of Endocrinology and Nephrology, Department of Medicine, Universität Leipzig, 04103 Leipzig, Germany; 37 Department of Internal Medicine, Erasmus Medical Centre, 3015 CE Rotterdam, The Netherlands; 38 Interfaculty Institute for Genetics and Functional Genomics, University Medicine Greifswald, 17489 Greifswald, Germany

## Abstract

Type 2 diabetes (T2D) is a very common disease in humans. Here we conduct a meta-analysis of genome-wide association studies (GWAS) with ~16 million genetic variants in 62,892 T2D cases and 596,424 controls of European ancestry. We identify 139 common and 4 rare variants associated with T2D, 42 of which (39 common and 3 rare variants) are independent of the known variants. Integration of the gene expression data from blood (*n* = 14,115 and 2765) with the GWAS results identifies 33 putative functional genes for T2D, 3 of which were targeted by approved drugs. A further integration of DNA methylation (*n* = 1980) and epigenomic annotation data highlight 3 genes (*CAMK1D*, *TP53INP1*, and *ATP5G1*) with plausible regulatory mechanisms, whereby a genetic variant exerts an effect on T2D through epigenetic regulation of gene expression. Our study uncovers additional loci, proposes putative genetic regulatory mechanisms for T2D, and provides evidence of purifying selection for T2D-associated variants.

## Abstract (web summary)

GWAS have so far identified 129 loci associated with type 2 diabetes (T2D). Here, the authors meta-analyse three large T2D GWA studies which uncovers 42 additional loci, further prioritize 33 functional genes using eQTL and mQTL data and propose regulatory mechanisms for three putative T2D genes.

## Introduction

Type 2 diabetes (T2D) is a common disease with a worldwide prevalence that increased rapidly from 4.7% in 1980 to 8.5% in 2014^1^. It is primarily caused by insulin resistance (failure of the body's normal response to insulin) and/or insufficient insulin production by beta cells^2^. Genetic studies using linkage analysis and candidate gene approaches have led to the discovery of an initial set of T2D-associated loci (e.g., *PPARG* and *TCF7L2*)^3,4^. Over the past decade, genome-wide association studies (GWAS) with increasing sample sizes have identified 144 genetic variants (not completely independent) at 129 loci associated with T2D^5,6^.

Despite a large number of variants discovered using GWAS, the associated variants in total, explains only a small proportion (~10%) of the heritability of T2D^7^. This well-known “missing heritability” problem is likely due to the presence of common variants (minor allele frequencies or MAF ≥ 0.01) that have small effects and have not yet been detected and/or rare variants that are not well tagged by common single nucleotide polymorphisms (SNPs)^7^. The contribution of rare variants to genetic variation in the occurrence of common diseases is under debate^8^, and a recent study suggested that the contribution of rare variants to the heritability of T2D is likely to be limited^9^. If most T2D-associated genetic variants are common in the population, continual discoveries of variants with small effects are expected from large-scale GWAS using the current experimental design. Furthermore, limited progress has been made in understanding the regulatory mechanisms of the genetic loci identified by GWAS. Thus, the etiology and the genetic basis underlying the development of this disease remain largely unknown. Recent methodological advances have provided us with an opportunity to identify functional genes and their regulatory elements by combining GWAS summary statistics with data from molecular quantitative trait loci studies with large sample sizes^10,11^.

In this study, we perform a meta-analysis of GWAS in a very large sample of T2D (62,892 cases and 596,424 controls), by combining 3 GWAS data sets of European ancestry: DIAbetes Genetics Replication and Meta-analysis (DIAGRAM)^5^, Genetic Epidemiology Research on Aging (GERA)^12^, and the full cohort release of the UK Biobank (UKB)^13^. We then integrate the GWAS meta-analysis results with gene expression and DNA methylation data to identify genes that might be functionally relevant to T2D and to infer plausible mechanisms, whereby genetic variants affect T2D risk through gene regulation by DNA methylation^11^. We further estimate the genetic architecture of T2D using whole-genome estimation approaches. Our study identifies additional T2D-risk variants, prioritizes functional genes, and proposes putative genetic regulatory mechanisms for T2D.

## Results

### Meta-analysis identifies 39 previously unknown loci

**Fig. 1:**
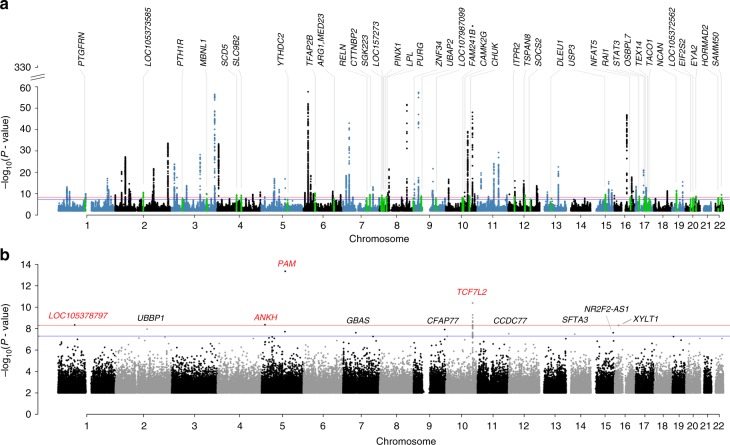
Manhattan plots of common- and rare-variant associations for T2D. **a** GWAS results for common variants (MAF ≥ 0.01) in the meta-analysis. The 39 novel loci are annotated and highlighted in green. **b** GWAS results of rare variants (0.0001 ≤ MAF < 0.01) in UKB. Four loci with *P* < 5 × 10^−9^ are highlighted in red. The blue lines denote the genome-wide significant threshold of *P* < 5 × 10^−8^, and the red lines denote a more stringent threshold of *P* < 5 × 10^−9^

We meta-analyzed 5,053,015 genotyped or imputed autosomal SNPs (MAF ≥ 0.01) in 62,892 T2D cases and 596,424 controls from the DIAGRAM (12,171 cases vs. 56,862 controls in stage 1 and 22,669 cases vs. 58,119 controls in stage 2), GERA (6905 cases and 46,983 controls) and UKB (21,147 cases and 434,460 controls) data sets after quality controls (Supplementary Fig. 1 and Methods). Summary statistics in DIAGRAM were imputed to the 1000 Genomes Project^14^ (1KGP) phase 1 using a summary data-based imputation approach, ImpG^15^ (Supplementary Note 1), and we used an inverse-variance method^16^ to meta-analyze the imputed DIAGRAM data with the summary data from GWAS analyses of GERA and UKB (Methods and Fig. 1a). We demonstrated by linkage disequilibrium (LD) score regression analysis^17,18^ that the inflation in test statistics due to population structure was negligible in each data set, and there was no evidence of sample overlap among the 3 data sets (Supplementary Note 2 and Supplementary Table 1). The mean *χ*^2^ statistic was 1.685. LD score regression analysis of the meta-analysis summary statistics showed an estimate of SNP-based heritability $\left( {\hat h_{{\mathrm{SNP}}}^2} \right)$ on the liability scale of 0.196 (s.e. = 0.011) and an estimate of intercept of 1.049 (s.e. = 0.014), consistent with a model in which the genomic inflation in test statistics is driven by polygenic effects^17^. After clumping the SNPs using LD information from the UKB genotypes (clumping *r*^2^ threshold = 0.01 and window size = 1 Mb), there were 139 near-independent variants at *P* < 5 × 10^−8^ (Supplementary Data 1). All of the loci previously reported by DIAGRAM were still genome-wide significant in our meta-analysis results. The most significant association was at rs7903146 (*P* = 1.3 × 10^−347^) at the known *TCF7L2* locus^4,19^. Among the 139 variants, 39 are not in LD with the known variants (Fig. 1 and Table 1). The result remained unchanged when the GERA cohort was imputed to Haplotype Reference Consortium (HRC) (Supplementary Fig. 2). We regarded these 39 variants as novel discoveries; more than half of them passed a more stringent significance threshold at *P* < 1 × 10^−8^ (Table 1), a conservative control of genome-wide false-positive rate (GWFPR) suggested by a recent simulation study^20^. The functional relevance of some novel gene loci to the disease was supported by existing biological or molecular evidence related to insulin and glucose (Supplementary Note 3). Forest plots showed that the effect directions of the 39 novel loci were consistent across the 3 GWAS data sets (Supplementary Fig. 3). Regional association plots showed that some loci have complicated LD structures, and it is largely unclear which genes are responsible for the observed SNP-T2D associations (Supplementary Fig. 4). We also performed gene-based analysis by GCTA-fastBAT^21^, and conditional analysis by GCTA-COJO^22^, and discovered 4 loci with multiple independent signals associated with T2D (Supplementary Notes 4–5, Supplementary Fig. 5, and Supplementary Data 2–4). Polygenic-risk score analysis showed high classification accuracy using SNPs effects estimated from the meta-analysis (Supplementary Note 6 and Supplementary Table 2). We further applied a stratified LD score regression method^23^ to dissect the SNP-based heritability into the contributions from SNPs in different functional annotation categories and cell types (Supplementary Note 7, Supplementary Figs. 6, 7, Supplementary Data 5, and Supplementary Table 3).

Of all the 139 T2D-associated loci identified in our meta-analysis, 16 and 25 were significant in insulin secretion and sensitivity GWAS, respectively, from the MAGIC consortium^24,25^ (see URLs section) after correcting for multiple tests (i.e., 0.05/139), with only 1 locus showing significant associations with both insulin secretion and sensitivity. The limited number of overlapping associations observed might be due to the relatively small sample sizes in the insulin studies. We further estimated the genetic correlation (*r*_g_) between insulin secretion (or sensitivity) and T2D by the bivariate LD score regression approach^18^ using summary-level data. The estimate of *r*_g_ between T2D and insulin secretion was −0.15 (s.e. = 0.10), and that between T2D and insulin sensitivity was −0.57 (s.e. = 0.10). Gene set enrichment test also showed that T2D-associated loci were enriched in “glucose homeostasis” and “insulin secretion” pathways (Supplementary Note 7, Supplementary Fig. 8, and Supplementary Data 6–7).

**Table 1:** Common variants at 39 previously unknown T2D-associated loci

CHR	BP	SNP	A1	A2	MAF	OR (95% CI)	*P*_GWAS_	Nearest gene
1	117530507	rs1127655	C	T	0.47	1.04 (1.03–1.06)	2.47E−08	*PTGFRN*
2	121309759	rs12617659	T	C	0.15	0.93 (0.91–0.95)	2.83E−11	*LOC105373585* (*GLI2*)
3	46925539	rs11926707	T	C	0.37	0.95 (0.94–0.97)	1.69E−08	*PTH1R*
3	152053250	rs4472028	T	C	0.44	1.05 (1.03–1.06)	2.08E−10	*MBNL1*
4	83584496	rs993380	A	G	0.33	1.05 (1.04–1.07)	4.59E−10	*SCD5*
4	103988899	rs7674212	T	G	0.41	0.95 (0.94–0.97)	6.18E−10	*SLC9B2*
5	112927686	rs10077431	A	C	0.21	0.95 (0.94–0.97)	4.76E−08	*YTHDC2*
6	50816887	rs72892910	T	G	0.17	1.07 (1.05–1.09)	6.43E−11	*TFAP2B*
6	131898208	rs2246012	C	T	0.16	1.05 (1.03–1.07)	2.43E−08	*ARG1*, *MED23*
7	103418846	rs2299383	T	C	0.42	1.04 (1.03–1.06)	1.49E−08	*RELN*
7	117510621	rs13239186	T	C	0.30	1.06 (1.04–1.07)	2.70E−10	*CTTNBP2*
8	8168987	rs7841082	T	C	0.44	0.96 (0.94–0.97)	4.94E−08	*SGK223*
8	9188762	rs11774915	T	C	0.34	1.05 (1.03–1.07)	8.73E−09	*LOC157273* (*TNKS*)
8	10633159	rs10100265	A	C	0.39	1.05 (1.03–1.07)	6.29E−10	*PINX1*
8	19852310	rs17411031	G	C	0.26	0.96 (0.94–0.97)	3.04E−08	*LPL*
8	30863722	rs10087241	G	A	0.41	1.05 (1.03–1.07)	2.80E−09	*PURG*
8	146003567	rs2294120	G	A	0.46	0.96 (0.94–0.97)	1.62E−08	*ZNF34*
9	34025640	rs1758632	C	G	0.38	0.95 (0.94–0.97)	1.36E−09	*UBAP2*
9	96919182	rs10114341	C	T	0.44	0.96 (0.95–0.97)	1.15E−08	*LOC107987099* (*PTPDC1*)
10	71469514	rs2616132	A	G	0.47	1.05 (1.03–1.06)	6.58E−09	*FAM241B*
10	75594050	rs2633310	T	G	0.44	0.96 (0.94–0.97)	2.38E−08	*CAMK2G*
10	101976501	rs11591741	C	G	0.44	0.95 (0.94–0.97)	1.23E−09	*CHUK*
12	26463082	rs11048456	C	T	0.24	1.05 (1.03–1.07)	2.97E−09	*ITPR2*
12	71439589	rs7138300	C	T	0.44	1.05 (1.03–1.06)	5.65E−10	*TSPAN8*
12	93978504	rs11107116	T	G	0.22	1.05 (1.03–1.07)	3.75E−08	*SOCS2*
13	51096095	rs963740	T	A	0.29	0.95 (0.94–0.97)	2.23E−08	*DLEU1*
15	63823301	rs982077	A	G	0.43	1.05 (1.03–1.06)	2.58E−10	*USP3*
16	69666683	rs244415	A	G	0.41	0.95 (0.94–0.97)	3.88E−09	*NFAT5*
17	17653411	rs12945601	T	C	0.39	1.05 (1.03–1.07)	1.72E−09	*RAI1*
17	40542501	rs17405722	A	G	0.07	1.09 (1.06–1.12)	2.28E−09	*STAT3*
17	45885756	rs9911983	C	T	0.43	0.96 (0.95–0.97)	4.82E−08	*OSBPL7*
17	56757584	rs302864	A	G	0.09	1.07 (1.05–1.10)	2.46E−08	*TEX14*
17	61687600	rs17631783	T	C	0.26	0.95 (0.94–0.97)	3.95E−08	*TACO1*
19	19407718	rs10401969	C	T	0.08	1.10 (1.07–1.13)	4.13E−12	*SUGP1*
20	22435749	rs6515236	C	A	0.25	0.95 (0.93–0.97)	3.34E−08	*LOC105372562* (*FOXA2*)
20	32675727	rs6059662	A	G	0.34	0.96 (0.94–0.97)	1.51E−08	*EIF2S2*
20	45594711	rs6066138	A	G	0.28	0.95 (0.94–0.97)	1.93E−09	*EYA2*
22	30552813	rs16988333	G	A	0.09	0.93 (0.90–0.95)	9.17E−09	*HORMAD2*
22	44377442	rs4823182	G	A	0.34	1.05 (1.03–1.07)	3.36E−10	*SAMM50*

CHR: chromosome, BP: base pair position in build hg19, A1: minor allele, A2: major allele, MAF: minor allele frequency, OR; odds ratio for A1, *P*_GWAS_: association *p* value from the GWAS meta-analysis, Nearest gene: if the nearest gene (within 1 Mb) is uncharacterized, a nearest characterized gene is shown in a bracket

### Rare variants associated with T2D

Very few rare variants-associated with T2D have been identified in previous studies^26–28^. We included 10,849,711 rare variants (0.0001 ≤ MAF < 0.01) in the association analysis in UKB and detected 11 rare variants at *P* < 5 × 10^−8^ and 4 of them were at *P* < 5 × 10^−9^ (Fig. 1b and Supplementary Table 4). We focused only on the 4 signals at *P* < 5 × 10^−9^ because a recent study suggested that a *P* value threshold of 5 × 10^−9^ is required to control a GWFPR at 0.05 in GWAS, including both common and rare variants imputed from a fully sequenced reference^20^. Three of the rare variants were located at loci with significant common variant associations. Variant rs78408340 (odds ratio (OR) = 1.33, *P* = 4.4 × 10^−14^) is a missense variant that encodes a *p.Ser539Trp* alteration in *PAM* and was reported to be associated with decreased insulin release from pancreatic beta cells^27^. Variant rs146886108 (OR = 0.72, *P* = 4.4 × 10^−9^), which showed a protective effect against T2D, is a novel locus and a missense variant that encodes p.Arg187Gln in *ANKH*^29^. Variant rs117229942 (OR = 0.70, *P* = 4.0 × 10^−11^) is an intron variant in *TCF7L2*^4^. Variant rs527320094 (OR = 2.74, *P* = 4.6 × 10^−9^), located in *LOC105378797*, is also a novel rare-variant association, with no other significant SNP (either common or rare) within a ±1 Mb window. We did not observe any substantial difference in association signals for these 4 variants between the results from BOLT-LMM^30^ and logistic regression^31^ considering the difference in sample size (Supplementary Table 4).

### Gene expression and DNA methylation associated with T2D

Most previous studies have reported the gene in closest physical proximity to the most significant SNP at a GWAS locus. However, gene regulation can be influenced by genetic variants that are physically distal to the genes^32^. To prioritize genes identified through the genome-wide significant loci that are functionally relevant to the disease, we performed a summary data-based Mendelian randomization (SMR) analysis^33^ using the top-associated expression quantitative trait locus (eQTL) as an instrumental variable to test for association between the expression level of each gene and T2D (Methods). We used GWAS summary data from our meta-analysis and eQTL summary data from the eQTLGen (*n* = 14,115) and CAGE consortia (*n* = 2765)^34^ for the SMR analysis (Methods). We identified 40 genes in eQTLGen and 24 genes in CAGE at an experimental-wise significance level (*P*_SMR_ < 2.7 × 10^−6^, i.e., 0.05/*m*_SMR_, with $m_{{\mathrm{SMR}}} = 18,602$ being the total number of SMR tests in the 2 data sets) (Supplementary Data 8–9). To filter out the SMR associations due to linkage (i.e., 2 causal variants in LD, one affecting gene expression and the other affecting T2D risk), all the significant SMR associations were followed by a HEterogeneity In Dependent Instruments (HEIDI)^33^ analysis to test whether there is heterogeneity in SMR estimates at SNPs in LD with the top-associated cis-eQTL (Methods). Therefore, genes not rejected by HEIDI (i.e., no evidence of heterogeneity) were those associated with T2D through pleiotropy at a shared genetic variant. Of the genes that passed the SMR test, 27 genes in eQTLGen and 15 genes in CAGE were not rejected by the HEIDI test (*P*_HEIDI_ > 7.8 × 10^−4^, i.e., 0.05/*m*_SMR_, with $m_{{\mathrm{SMR}}} = 64$ being the total number of SMR tests in the 2 data sets) (Tables 2–3 and Supplementary Data 8–9), with 7 genes in common and 33 unique genes in total. SNPs associated with the expression levels of genes including *EHHADH* (rs7431357), *SSSCA1* (rs1194076), and *P2RX4* (rs2071271) in eQTLGen were not significant in the T2D meta-analysis, likely due to the lack of power; these SNPs were expected to be detected in future studies with larger sample sizes.

**Table 2:** Putative functional genes for T2D identified from the SMR analysis in eQTLGen

probe ID	Chr	Gene	topSNP	A1	A2	Freq	*P*_GWAS_	*P*_eQTL_	*P*_SMR_	*P*_HEIDI_
55879	1	*CD101*	rs10737727	C	A	0.48	1.1E−07	1.2E−116	2.5E−07	9.2E−03
68011	2	*CEP68*	rs2249105	G	A	0.38	4.1E−10	1.3E−190	1.0E−09	2.9E−02
9391	3	*EHHADH*	rs7431357	A	G	0.16	2.4E−07	1.6E−39	1.4E−06	1.2E−01
43929	4	*RP11-10L12.4*	rs223359	T	C	0.48	1.2E−07	<1E−300	1.4E−07	3.1E−02
68382	5	*ANKH*	rs1061813	G	A	0.46	3.4E−09	1.4E−110	1.3E−08	3.9E−01
62965	5	*POC5*	rs10515213	G	A	0.21	2.1E−06	1.3E−244	2.5E−06	9.4E−04
40809	6	*RREB1*	rs2714337	T	A	0.35	3.9E−10	2.8E−48	1.0E−08	1.6E−03
44795	6	*MICB*	rs2253042	T	C	0.33	2.1E−08	<1E−300	2.0E−08	8.8E−04
29725	6	*HLA-DQB1*	rs1063355	T	G	0.43	3.7E−19	1.5E−38	1.6E−13	7.6E−03
12660	6	*CENPW*	rs1591805	G	A	0.51	1.6E−09	1.4E−21	3.8E−07	3.2E−02
56635	6	*ARG1*	rs2246012	C	T	0.15	2.4E−08	<1E−300	2.7E−08	9.0E−01
39116	6	*MED23*	rs3756784	G	T	0.19	2.6E−08	6.9E−67	1.3E−07	8.1E−01
16667	8	*TP53INP1*	rs10097617	C	T	0.51	7.5E−08	9.9E−86	2.4E−07	2.5E−01
17817	8	*RPL8*	rs2958517	G	A	0.47	1.5E−06	<1E−300	1.8E−06	7.0E−01
51129	10	*CAMK1D*	rs11257655	T	C	0.20	2.0E−17	<1E−300	1.1E−16	2.3E−02
45148	10	*CAMK1D*	rs11257655	T	C	0.20	2.0E−17	3.7E−131	1.2E−15	2.6E−02
51050	10	*CAMK1D*	rs11257655	T	C	0.20	2.0E−17	<1E−300	1.3E−16	1.5E−02
14584	10	*CAMK1D*	rs11257655	T	C	0.20	2.0E−17	<1E−300	1.2E−16	4.2E−03
55828	10	*CWF19L1*	rs34027394	A	G	0.42	5.2E−09	<1E−300	6.4E−09	4.7E−01
54041	10	*SNORA12*	rs34762508	T	C	0.42	5.8E−09	1.3E−16	1.9E−06	9.1E−01
564	10	*PLEKHA1*	rs11200629	G	A	0.48	5.1E−08	5.0E−151	1.1E−07	1.4E−01
44452	10	*PLEKHA1*	rs7072204	G	A	0.48	5.4E−08	1.8E−180	1.1E−07	1.5E−01
54567	11	*SSSCA1*	rs1194076	A	C	0.24	7.6E−07	1.4E−268	9.3E−07	8.5E−01
59012	11	*ARAP1*	rs9667947	C	T	0.15	2.1E−20	2.0E−10	1.5E−07	5.4E−03
64698	12	*P2RX4*	rs2071271	T	C	0.27	3.6E−07	<1E−300	4.5E−07	2.9E−01
14501	12	*CAMKK2*	rs11065504	C	G	0.36	2.0E−06	<1E−300	2.4E−06	4.3E−03
25086	12	*CAMKK2*	rs11065504	C	G	0.36	2.0E−06	<1E−300	2.4E−06	2.2E−03
19328	15	*C15orf38*	rs7174878	A	G	0.26	5.2E−10	2.5E−214	1.0E−09	3.0E−03
55328	15	*RCCD1*	rs2290202	T	G	0.14	2.3E−07	<1E−300	2.9E−07	2.8E−03
28542	17	*ANKFY1*	rs4790598	G	T	0.38	7.1E−08	1.8E−45	4.5E−07	1.1E−02
9982	17	*ATP5G1*	rs1962412	T	C	0.31	5.6E−11	1.1E−120	2.9E−10	2.6E−03
42278	17	*ATP5G1*	rs318095	T	C	0.48	4.0E−12	3.6E−117	3.9E−11	5.2E−02
60420	17	*UBE2Z*	rs15563	A	G	0.48	3.4E−12	1.3E−52	2.6E−10	4.7E−03
60551	17	*UBE2Z*	rs962272	A	G	0.48	3.8E−12	9.6E−67	1.4E−10	7.4E−02

Columns are probe ID, probe chromosome, gene name, probe position, SNP name, SNP position, effect allele, other allele, frequency of the effect allele in the reference sample, GWAS *P* value, eQTL *P* value, SMR *P* value and HEIDI *P* value

**Table 3:** Putative functional genes for T2D identified from the SMR analysis in CAGE

probe ID	Chr	Gene	topSNP	A1	A2	Freq	*P*_GWAS_	*P*_eQTL_	*P*_SMR_	*P*_HEIDI_
ILMN_1754865	1	*PABPC4*	rs1985076	C	T	0.22	2.0E−12	3.0E−23	8.9E−09	4.1E−01
ILMN_1757343	1	*PABPC4*	rs17513135	T	C	0.23	2.7E−13	7.7E−32	6.3E−10	3.1E−01
ILMN_1795464	6	*LTA*	rs2516479	G	C	0.40	3.9E−10	9.4E−28	5.9E−08	5.6E−03
ILMN_1712390	6	*CUTA*	rs115196245	C	G	0.03	5.1E−10	1.2E−27	6.7E−08	1.1E−02
ILMN_1812281	6	*ARG1*	rs2246012	C	T	0.15	2.4E−08	1.1E−113	5.3E−08	8.6E−01
ILMN_1714108	8	*TP53INP1*	rs896853	G	C	0.48	1.3E−07	2.3E−33	1.3E−06	4.8E−01
ILMN_1711314	10	*NUDT5*	rs11257655	T	C	0.20	2.0E−17	8.0E−36	2.4E−12	2.8E−03
ILMN_1795561	10	*CAMK1D*	rs11257655	T	C	0.20	2.0E−17	2.7E−112	2.2E−15	1.6E−01
ILMN_1751561	10	*CAMK1D*	rs11257655	T	C	0.20	2.0E−17	8.6E−102	3.3E−15	8.4E−02
ILMN_1906187	10	*LOC283070*	rs11257655	T	C	0.20	2.0E−17	1.9E−101	3.4E−15	6.9E−03
ILMN_1651886	10	*CWF19L1*	rs34027394	A	G	0.42	5.2E−09	3.0E−130	1.4E−08	4.8E−01
ILMN_1662839	10	*PLEKHA1*	rs11200594	C	T	0.52	1.1E−07	1.8E−44	6.2E−07	1.9E−01
ILMN_1727134	12	*KLHDC5*	rs12578595	T	C	0.20	1.9E−11	9.9E−25	1.7E−08	3.3E−03
ILMN_1813846	12	*P2RX4*	rs2071271	T	C	0.27	3.6E−07	2.1E−68	1.1E−06	2.7E−01
ILMN_1743021	12	*CAMKK2*	rs35898441	T	C	0.35	4.1E−07	9.9E−136	7.5E−07	1.3E−02
ILMN_2367638	12	*CAMKK2*	rs3794207	T	C	0.35	6.5E−07	4.0E−132	1.2E−06	2.6E−02
ILMN_2189406	15	*C15orf38*	rs12594774	A	G	0.26	2.7E−10	4.9E−28	3.8E−08	1.1E−02
ILMN_1712430	17	*ATP5G1*	rs7212779	A	G	0.29	1.6E−10	7.7E−26	4.7E−08	1.5E−02
ILMN_1676393	17	*ATP5G1*	rs12325727	G	A	0.52	6.3E−11	1.1E−31	1.3E−08	2.7E−01

Columns are probe ID, probe chromosome, gene name, probe position, SNP name, SNP position, effect allele, other allele, frequency of the effect allele in the reference sample, GWAS *P* value, eQTL *P* value, SMR *P* value, and HEIDI *P* value

To identify the regulatory elements associated with T2D risk, we performed SMR analysis using methylation quantitative trait locus (mQTL) data from McRae et al.^35^ (*n* = 1980) to identify DNA methylation (DNAm) sites associated with T2D through pleiotropy at a shared genetic variant. In total, 235 DNAm sites were associated with T2D, with *P*_SMR_ < 6.3 × 10^−7^ $\left( {m_{{\mathrm{SMR}}} = 78,961} \right)$ and *P*_HEIDI_ > 1.6 × 10^−4^ $\left( {m_{{\mathrm{HEIDI}}} = 323} \right)$ (Supplementary Data 10); these DNAm sites were significantly enriched in promoters (fold change = 1.60, *P*_enrichment_ = 1.6 × 10^−7^) and weak enhancers (fold change = 1.74, *P*_enrichment_ = 1.4 × 10^−2^) (Supplementary Note 8 and Supplementary Fig. 9). Identification of DNAm sites and their target genes relies on consistent association signals across omics levels^11^. To demonstrate this, we conducted the SMR analysis to test for associations between the 235 T2D-associated DNAm sites and the 33 T2D-associated genes and identified 22 DNAm sites associated with 16 genes in eQTLGen (Supplementary Data 11) and 21 DNAm sites associated with 15 genes in CAGE (Supplementary Data 12) at *P*_SMR_ < 2.5 × 10^−7^ $\left( {m_{{\mathrm{SMR}}} = 202,609} \right)$ and *P*_HEIDI_ > 2.1 × 10^−4^ $\left( {m_{{\mathrm{HEIDI}}} = 235} \right)$. These results can be used to infer plausible regulatory mechanisms for how genetic variants affect T2D risk by regulating the expression levels of genes through DNAm (see below).

### SMR associations in multiple T2D-relevant tissues

To replicate the SMR associations in a wider range of tissues relevant to T2D, we performed SMR analyses based on cis-eQTL data from 4 tissues in GTEx^36^ (i.e., adipose subcutaneous tissue, adipose visceral omentum, liver, and pancreas). We denoted these 4 tissues as GTEx-AALP. Of the 27 putative T2D genes identified by SMR and HEIDI using the eQTLGen data, 10 had a cis-eQTL at *P*_eQTL_ < 5 × 10^−8^ in at least one of the 4 GTEx-AALP tissues (Supplementary Data 13). Note that the decrease in eQTL detection power is expected given the much smaller sample size of GTEx-AALP (*n* = 153–385) compared to that of eQTLGen (*n* = 14,115), as demonstrated by simulation (Supplementary Note 9 and Supplementary Fig. 10). As a benchmark, 17 of the 27 genes had a cis-eQTL at *P*_eQTL_ < 5 × 10^−8^ in GTEx-blood (*n* = 369). We first performed the SMR analysis in GTEx-blood and found that 12 of the 17 genes were replicated at *P*_SMR_ < 2.9 × 10^−3^ (i.e., 0.05/17) (Supplementary Data 13), an expected high replication rate given the simulation result (Supplementary Fig. 10). We then conducted the SMR analysis in GTEx-AALP. The result showed that 8 of the 10 genes showed significant SMR associations at *P*_SMR_ < 1.3 × 10^−3^ (i.e., 0.05/40) in at least one of the 4 GTEx-AALP tissues, a replication rate comparable to that found in GTEx-blood. Among the 8 genes, *CWF19L1*, for which the cis-eQTL effects are highly consistent across different tissues, was significant in all the data sets (Supplementary Fig. 11).

The replication analysis described above depends heavily on the sample sizes of eQTL studies. A less sample-size-dependent approach is to quantify how well the effects of the top associated cis-eQTLs for all the 27 putative T2D genes estimated in blood (i.e., the eQTLGen data) correlate with those estimated in the GTEx tissues, accounting for sampling variation in estimated SNP effects^37^. This approach avoids the need to use a stringent *P* value threshold to select cis-eQTLs in the GTEx tissues with small sample sizes. We found that the mean correlation of cis-eQTL effects between eQTLGen blood and GTEx-AALP was 0.47 (s.e. = 0.16), comparable to and not significantly different from the value of 0.64 (s.e. = 0.16) between eQTLGen and GTEx-blood. We also found that the estimated SMR effects of 18 genes, which passed the SMR test and were not rejected by the HEIDI test in either eQTLGen or GTEx, were highly correlated (Pearson’s correlation *r* = 0.80) (Supplementary Fig. 12). Note that this correlation is not expected to be unity because of differences in the technology used to measure gene expression (Illumina gene expression arrays for eQTLGen vs. RNA-seq for GTEx). We also performed co-localization analyses using COLOC^38^, a Bayesian approach to seek evidence of a locus associated with two traits. We found that most of the genes that passed the genome-wide significant threshold in the SMR test also had extremely high posterior probabilities of associations with T2D from the COLOC analysis (Supplementary Fig. 13).

These results support the validity of using eQTL data from blood for the SMR and HEIDI analysis; using this method, we can make use of eQTL data from very large samples to increase the statistical power, consistent with the conclusions of a recent study^37^. In addition, tissue-specific effects that are not detected in blood will affect the power of the SMR and HEIDI analysis rather than generating false positive associations.

### Putative regulatory mechanisms for 3 T2D genes

Here, we used the genes *CAMK1D*, *TP53INP1*, and *ATP5G1* as examples to hypothesize possible mechanisms of how genetic variants affect T2D risk by controlling DNAm for gene regulation^11^. Functional gene annotation information was acquired from the Roadmap Epigenomics Mapping Consortium (REMC)^39^.

**Fig. 2:**
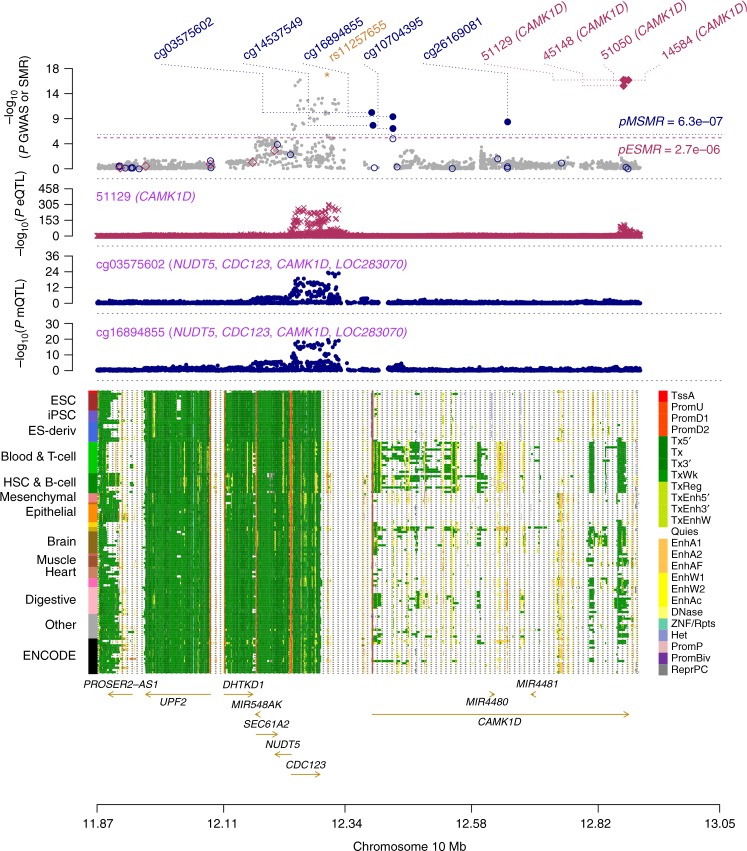
Prioritizing genes and regulatory elements at the *CAMK1D* locus for T2D. The results of the SMR analysis that integrates data from GWAS, eQTL, and mQTL studies are shown. The top plot shows −log_10_(*P* value) of SNPs from the GWAS meta-analysis for T2D. Red diamonds and blue circles represent −log_10_(*P* value) from the SMR tests for associations of gene expression and DNAm probes with T2D, respectively. Solid diamonds and circles represent the probes not rejected by the HEIDI test. The yellow star denotes the top cis-eQTL SNP rs11257655. The second plot shows −log_10_(*P* value) of the SNP association for gene expression probe 51129 (tagging *CAMK1D*). The third plot shows −log_10_(*P* value) of the SNP association with DNAm probes cg03575602 and cg16894855 from the mQTL study. The bottom plot shows 25 chromatin state annotations (indicated by colors) of 127 samples from Roadmap Epigenomics Mapping Consortium (REMC) for different primary cells and tissue types (rows)

**Fig. 3:**
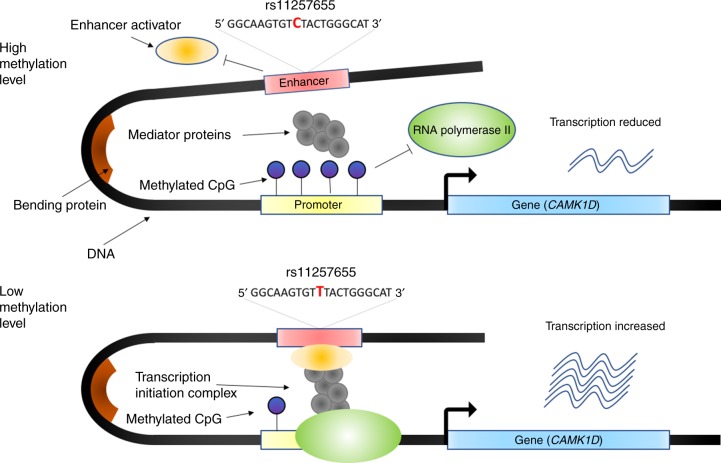
Hypothesized regulatory mechanism at the *CAMK1D* locus for T2D. When the allele of rs11257655 in the enhancer region (red) changes from C to T, the enhancer activator protein *FOXA1*/*FOXA2* (orange ellipsoid) binds to the enhancer region and the DNA methylation level in the promoter region is reduced; this increases the binding efficiency of RNA polymerase II recruited by mediator proteins (gray circles) and, therefore increases the transcription of *CAMK1D*

The significant SMR association of *CAMK1D* with T2D was identified in both eQTL data sets (Tables 2–3 and Supplementary Data 8–9). The top eQTL, rs11257655, located in the intergenic region (active enhancer) between *CDC123* and *CAMK1D*, was also a genome-wide significant SNP in our meta-analysis (*P* = 2.0 × 10^−17^). It was previously shown that rs11257655 is located in the binding motif for *FOXA1*/*FOXA2* and that the T allele of this SNP is a risk allele that increases the expression level of *CAMK1D* through allelic-specific binding of *FOXA1* and *FOXA2*^40^. Another functional study demonstrated that increasing the expression of *FOXA1* and its subsequent binding to enhancers was associated with DNA demethylation^41^. Our analysis was consistent with previous studies in showing that the T allele of rs11257655 increases both *CAMK1D* transcription ($\hat \beta = 0.553$, s.e. = 0.014, where $\beta$ is the allele substitution effect on gene expression in standard deviation units) and T2D risk (OR = 1.076, s.e. = 0.009) (Supplementary Data 8, 9, and 11). Moreover, rs11257655 was also the top mQTL (Fig. 2); the T allele of this SNP is associated with decreased methylation at the site cg03575602 in the promoter region of *CAMK1D*, suggesting that the T allele of rs11257655 up-regulates the transcription of *CAMK1D* by reducing the methylation level at cg03575602. Leveraging all the information above, we proposed the following model of the genetic mechanism at *CAMK1D* for T2D risk (Fig. 3). In the presence of the T allele at rs11257655, *FOXA1*/*FOXA2* and other transcription factors bind to the enhancer region and form a protein complex that leads to a decrease in the DNAm level of the promoter region of *CAMK1D* and recruits the RNA polymerase to the promoter, resulting in an increase in the expression of *CAMK1D* (Fig. 3). A recent study showed that the T risk allele is correlated with reduced DNAm and increased chromatin accessibility across multiple islet samples^42^ and that it is associated with disrupted beta cell function^43^. Our inference highlights the role of promote–enhancer interaction in gene regulation, analytically indicated by the integrative analysis using the SMR and HEIDI approaches.

**Fig. 4:**
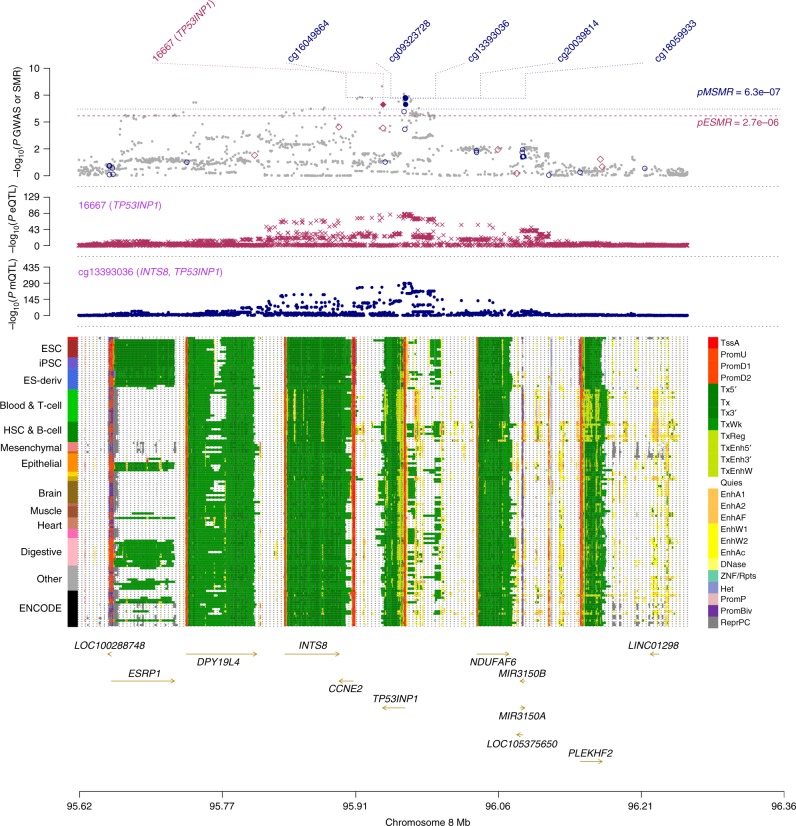
Prioritizing genes and regulatory elements at *TP53INP1* locus for T2D. Shown are the results from the SMR analysis that integrates data from GWAS, eQTL, and mQTL studies. The top plot shows −log_10_(*P* value) from the GWAS meta-analysis for T2D. Red diamonds and blue circles represent −log_10_(*P* value) from the SMR tests for associations of gene expression and DNAm probes with T2D, respectively. Solid diamonds and circles represent the probes not rejected by the HEIDI test. The second plot shows −log_10_(*P* value) of the SNP association with gene expression probe 16667 (tagging *TP53INP1*). The third plot shows −log_10_(*P* value) of the SNP association with DNAm probe cg13393036 and cg09323728. The bottom plot shows 25 chromatin state annotations (indicated by colors) of 127 samples from Roadmap Epigenomics Mapping Consortium (REMC) for different primary cells and tissue types (rows)

**Fig. 5:**
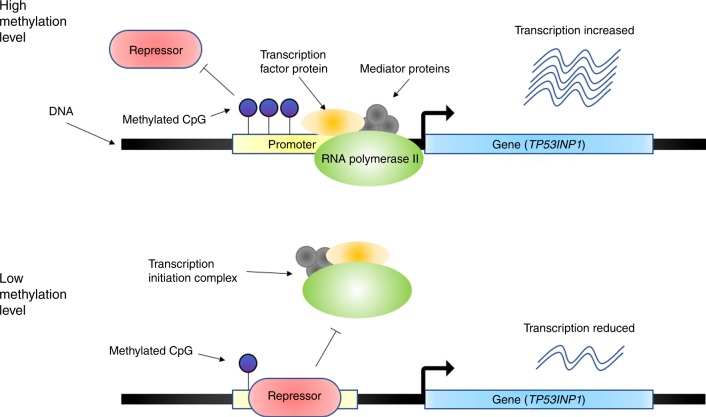
Hypothesized regulatory mechanism at the *TP53INP1* locus for T2D. When the promoter region is highly methylated, which prevents binding of repressor protein (red rounded rectangle) to the promoter region, RNA polymerase II (green ellipsoid), transcription factor protein (orange ellipsoid) and mediator proteins (gray circles) will form a transcription initiation complex that increases the transcription. However, when the methylation level of the promoter region is low, repressor protein can more efficiently bind to the promoter, blocking the binding of the transcription initiation complex to the promoter, which decreases the transcription of *TP53INP1*

The second example is *TP53INP1*, the expression level of which was positively associated with T2D as indicated by the SMR analysis (Table 2 and Supplementary Data 8). This was supported by previous findings that the protein encoded by *TP53INP1* regulated the *TCF7L2*-p53-p53INP1 pathway in such a way as to induce apoptosis and that the survival of pancreatic beta cells was associated with the level of expression of *TP53INP1*^44^. *TP53INP1* was mapped as the target gene for three DNAm sites (cg13393036, cg09323728, and cg23172400) by SMR (Fig. 4). All 3 DNAm sites were located in the promoter region of *TP53INP1* and had positive effects on the expression level of *TP53INP1* and on T2D risk (Supplementary Data 8, 10, and 11). Based on these results, we proposed the following hypothesis for the regulatory mechanism (Fig. 5). When the DNAm level of the promoter region is low, expression of *TP53INP1* is suppressed due to the binding of repressor(s) to the promoter. When the DNAm level of the promoter region is high, the binding of repressor(s) is disrupted, allowing the binding of transcription factors that recruit RNA polymerase and resulting in up-regulation of gene expression. Increased expression of this gene has been shown to increase T2D risk by decreasing the survival rate of pancreatic beta cells through a *TCF7L2*-p53-p53INP1-dependent pathway.

The third example involves 2 proximal genes, *ATP5G1* and *UBE2Z*, the expression levels of which were significantly associated with T2D according to the SMR analysis (Table 2 and Supplementary Data 8). A methylation probe (cg16584676) located in the promoter region of *UBE2Z* was associated with the expression levels of both *ATP5G1* and *UBE2Z* (Supplementary Fig. 14a), suggesting that these two genes are co-regulated by a genetic variant through DNAm. The effect of cg16584676 on gene expression was negative (Supplementary Data 11 and 12), implying the following plausible mechanism. A genetic variant near *ATP5G1* exerts an effect on T2D by increasing the DNAm levels of the promoters for *ATP5G1* and *UBE2Z*; this decreases the binding affinity of the transcription factors that recruit RNA polymerase, resulting in down-regulation of gene expression and ultimately leading to an increase in T2D risk (Supplementary Fig. 14b). *ATP5G1* has been shown to encode a subunit of mitochondrial ATP synthase, and *UBE2Z* is a ubiquitin-conjugating enzyme. Insulin receptors could be degraded by *SOCS* proteins during ubiquitin-proteasomal degradation, and *ATP5G1* and *UBE2Z* are likely to be involved in this pathway^45^. The function of insulin receptors is to regulate glucose homeostasis through the action of insulin and other tyrosine kinases, and dysfunction of these receptors leads to insulin resistance and increases T2D risk.

The 3 examples above provide hypotheses for how genetic variants may affect T2D risk through regulatory pathways and demonstrate the power of integrative analysis of omics data for this purpose. These examples describe putative candidates that could be prioritized in future functional studies.

### Potential drug targets

In the SMR analysis described above, we identified 33 putative T2D genes. We matched these genes in the DrugBank database (see URLs section) and found that 3 genes (*ARG1*, *LTA*, and *P2RX4*) are the targets of several approved drugs (drugs that have been approved in at least one jurisdiction). *ARG1* (UniProt ID: P05089), whose expression level was negatively associated with T2D risk, is targeted by three approved drugs: ornithine (DrugBank ID: DB00129), urea (DrugBank ID: DB03904), and manganese (DrugBank ID: DB06757), but the pharmacological mechanism of action of these drugs remains unknown. Arginase (*ARG1* is an isoform of arginase in liver) is a manganese-containing enzyme that catalyzes the hydrolysis of arginine to ornithine and urea. Arginase in vascular tissue might be a potential therapeutic target for the treatment of vascular dysfunction in diabetes^46^. Metformin, an oral antidiabetic drug that is used in the treatment of diabetes, was reported to increase *ARG1* expression in a murine macrophage cell line^47^, consistent with our SMR result that increased expression of *ARG1* was associated with decreased T2D risk (Supplementary Data 8). There was also evidence for an interaction between *ARG1* and metformin (Comparative Toxicogenomics Database, see URLs section). The likely mechanism is that metformin activates AMP-activated protein kinase (AMPK), resulting in increased expression of *ARG1*^48^, again consistent with our SMR result. *LTA* (UniProt ID: P08637), whose expression level was negatively associated with T2D risk, is targeted by the approved drug etanercept (DrugBank ID: DB00005) for rheumatoid arthritis (RA) treatment. *P2RX4* (UniProt ID: Q99571), the expression level of which was positively associated with T2D risk, is targeted by eslicarbazepine acetate (DrugBank ID: DB09119; antagonist for *P2RX4*). Eslicarbazepine acetate is an anticonvulsant that inhibits repeated neuronal firing and stabilizes the inactivated state of voltage-gated sodium channels; its pharmacological action makes it useful as an adjunctive therapy for partial-onset seizures^49^. Antagonists of *P2RX4* inhibit high glucose and are useful in the treatment of diabetic nephropathy^50^. We also explored whether any of these three genes have potential adverse effects by checking the associations of the lead variants at the three loci with lipid- and insulin-related traits from previous studies (Supplementary Note 10 and Supplementary Data 14). We further found two additional genes that are targeted by an approved veterinary drug and a nutraceutical drug, respectively (Supplementary Note 10).

### Natural selection of T2D-associated variants

**Fig. 6:**
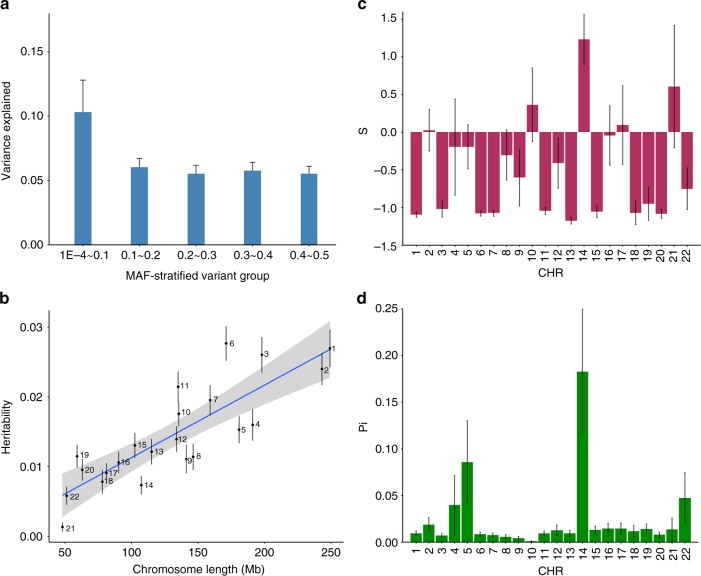
Estimation of the genetic architecture parameters for T2D in UKB. Shown in the panel **a** are the results from the GREML-LDMS analysis, and those in panels **b, c** and **d** are the results from the BayesS analysis using the UKB data. Error bars are standard errors of the estimates. **a** Variance explained by SNPs in each MAF bin. We combined the estimates of the first three bins (MAF < 0.1) to harmonize the width of all MAF bins. **b** Chromosome-wide SNP-based heritability against chromosome length. **c** Estimate of the BayesS parameter (*S*) reflecting the strength of purifying selection on each chromosome. **d** Proportion of SNPs with non-zero effects on each chromosome (*π*)

We performed an LD- and MAF-stratified GREML analysis^51^ (Methods) in a subset of unrelated individuals in UKB (*n* = 15,767 cases and 104,233 controls) to estimate the variance explained by SNPs in different MAF ranges (*m* = 18,138,214 in total). We partitioned the SNPs into 7 MAF bins with high- and low-LD bins within each MAF bin to avoid MAF- and/or LD-mediated bias in $\hat h_{{\mathrm{SNP}}}^2$ (Methods). The $\hat h_{{\mathrm{SNP}}}^2$ was 33.2% (s.e. = 2.1%) on the liability scale (Supplementary Table 5). Under an evolutionary neutral model and a constant population size^52^, the explained variance is uniformly distributed as a function of MAF, which means that the variance explained by variants with MAF ≤ 0.1 equals that explained by variants with MAF > 0.4. However, in our results, the MAF bin containing low-MAF and rare variants (MAF ≤ 0.1) showed a larger estimate than any other MAF bin (Fig. 6a and Supplementary Table 5), consistent with a model of negative (purifying) selection or population expansion^53^. To further distinguish between the two models (negative selection vs. population expansion), we performed an additional analysis using a recently developed method, BayesS^54^ (implemented in GCTB, see URLs section) to estimate the relationship between variance in effect size and MAF (Methods). The method also allowed us to estimate $\hat h_{{\mathrm{SNP}}}^2$ and polygenicity (*π*) on each chromosome. The results (Fig. 6b) showed that the$\hat h_{{\mathrm{SNP}}}^2$ of each chromosome was highly correlated with its length (Pearson’s correlation *r* = 0.92). The mean estimate of *π*, i.e., the proportion of SNPs with non-zero effects, was 1.75% across all chromosomes (Fig. 6c and Supplementary Table 6), suggesting a high degree of polygenicity for T2D. The sum of per-chromosome $\hat h_{{\mathrm{SNP}}}^2$ from BayesS was 31.9% (s.e. = 4.1%) on the liability scale, slightly higher than that based on HapMap3 SNPs from a Haseman-Elston regression analysis (28.7%, s.e. = 1.1%) using a full set of unrelated UKB individuals (*n* = 348,580) or from an LD score regression analysis (22.6%, s.e. = 1.2%) using all the UKB individuals (*n* = 455,607) (Supplementary Table 7). The variance in effect size was significantly negatively correlated with MAF ($\hat S$ = −0.53, s.e. = 0.09), consistent with a model of negative selection on deleterious rare alleles (Fig. 6d) and inconsistent with a recent study^9^ concluding that T2D-associated loci have not been under natural selection. Our conclusion regarding negative selection is also consistent with the observation that the minor alleles of 9 of the 11 rare variants at $P < 5 \times 10^{ - 8}$ were T2D risk alleles (Supplementary Table 4). The signal of negative selection implies that a large number of rare variants are expected to be discovered in future GWAS in which appropriate genotyping strategies are used.

## Discussion

In this study, we sought to identify novel genetic loci associated with T2D by a meta-analysis of GWAS with a very large sample size and to infer plausible genetic regulation mechanisms at known and novel loci by an integrative analysis of GWAS and omics data. We identified 139 near-independent common variants $\left( {P < 5 \times 10^{ - 8}} \right)$ and 4 rare variants $\left( {P < 5 \times 10^{ - 9}} \right)$ for T2D in the meta-analysis. Of the 139 common loci, 39 were novel compared with the results of all 49 previous T2D GWAS from the GWAS Catalog (see URLs section)^55^, including the 2 recent studies by DIAGRAM^56^ and Zhao et al.^57^. We did not detect evidence for sex or age heterogeneity in UKB (Supplementary Note 11, Supplementary Fig. 14, and Supplementary Table 8). By integrating omics data, we have inferred the genetic mechanisms for the 3 genes *CAMK1D*, *TP53INP1*, and *ATP5G1*; the inferred mechanisms suggest that enhancer-promoter interactions with DNA methylation play an important role in mediating the effects of genetic variants on T2D risk. These findings provide deeper insight into the etiology of T2D and suggest candidate genes for functional studies in the future. Furthermore, our estimation of genetic architecture suggests that T2D is a polygenic trait for which both rare and common variants contribute to the genetic variation and indicates that rarer variants tend to have larger effects on T2D risk (Fig. 6c and Supplementary Table 4). Assuming that most new mutations are deleterious for fitness, our result is consistent with a model in which mutations that have larger effects on T2D (and thereby on fitness through pleiotropy) are more likely to be maintained at low frequencies in the population by negative (purifying) selection.

This study has a number of limitations. First, the SNP-T2D associations identified by the meta-analysis might be biased by misdiagnosis of T1D (type 1 diabetes) and latent autoimmune diabetes in adults^58^. Previous studies found that biases in SNP-T2D associations due to misdiagnosis are likely to be very modest^5,56^. We showed by 2 additional analyses based on known T1D loci that most of the novel SNP-T2D associations identified in this study are unlikely to be driven by misdiagnosed T1D cases (Supplementary Note 12 and Supplementary Data 15). Second, some of the T2D-associated SNPs might confer T2D risk through mediators such as obesity or dyslipidemia. To explore this possibility, we performed a summary data-based conditional analysis of the 139 T2D-associated SNPs conditioning on body mass index (BMI) or dyslipidemia by GCTA-mtCOJO^59^ using GWAS data for these 2 traits from UKB. It appeared that the effect sizes of most T2D-associated SNPs, with the exception of a few outliers (e.g., *FTO*, *MC4R*, *POCS*, and *TFAP2B*), were not affected by BMI or dyslipidemia (Supplementary Fig. 16). These outliers were among those showing the strongest associations with BMI^60^. Third, among the 39 novel loci, there was only 1 locus (*ARG1*/*MED23*, Supplementary Fig. 17) at which the association between gene expression and T2D risk was significant in SMR and not rejected by HEIDI (Tables 2–3). This is because the power of the SMR test depends primarily on the SNP effect from GWAS^10^, which is small for the novel loci. Fourth, the sample sizes of eQTL data from the disease relevant tissues were relatively small. We used the eQTL data from blood to take advantage of the large sample sizes. This maximized the power for detecting genes for which the eQTL effects are consistent across tissues (Supplementary Fig. 10) but might have missed genes for which the eQTL effects are specific to the T2D-relevant tissues. Moreover, the pancreatic islets constitute only 1–2% of the whole pancreas volume^61^ and previous studies revealed islet-specific gene activity for T2D^62,63^. Therefore, in our SMR analysis using GTEx-pancreas data, genes with islet-specific transcription or eQTL effects could be missed. Finally, we employed the SMR and HEIDI methods to map CpG sites to their target genes and to identify the CpG sites associated with T2D because of pleiotropy. The SMR approach uses genome-wide significant mQTL as an instrumental variable for each CpG site, which requires a large sample size for the mQTL discovery. In this study, we used mQTL data based on Illumina HumanMethylation450 arrays because of the relatively large sample size (*n* = 1980). Unfortunately, we did not have access to mQTL data from whole-genome bisulfite sequencing (WGBS) in a large sample. Nevertheless, it is noteworthy that there are three T2D-associated variants at the *CAMK1D*/*CDC123*, *ADCY5*, and *KLHDC5* loci that show hypomethylation and allelic imbalance as identified by Thurner et al.^42^ using WGBS data (*n* = 10), all of which were genome-wide significant in our mQTL-based SMR analysis. In addition, a previous study showed that T2D-associated loci were enriched in islet stretch enhancers^63^, ~54.1% of which were tagged by at least one of the DNAm probe in the 450 K array (annotation data from ref. ^64^). Despite these limitations, our study highlights the benefits of integrating multiple omics data to identify functional genes and putative regulatory mechanisms driven by local genetic variation. Future applications of integrative omics data analyses are expected to improve our understanding of the biological mechanisms underlying T2D and other common diseases.

## Methods

### Summary statistics of DIAGRAM, GERA, and UKB

The data used in this study were derived from 659,316 individuals of European ancestry and a small cohort from Pakistan, and were obtained from three data sets: DIAbetes Genetics Replication And Meta-analysis (DIAGRAM)^5^, Genetic Epidemiology Research on Adult Health and Aging (GERA)^12^ and UKB^13^.

DIAGRAM: The DIAGRAM data were obtained from publicly available databases (see URLs section) and included 2 stages of summary statistics. In stage 1, there were 12,171 cases and 56,862 controls from 12 GWAS cohorts of European descent, and the genotype data were imputed to the HapMap2 Project^65^ (~2.5 million SNPs after quality control). In stage 2, there were 22,669 cases and 58,119 controls genotyped on Metabochips (~137,900 SNPs), including 1178 cases and 2472 controls of Pakistani descent. There was limited evidence of genetic heterogeneity between individuals of European and those of Pakistani descent for T2D^5^. The sample prevalence was 23.3% (17.6% in stage 1 and 28.1% in stage 2). We imputed the stage 1 summary statistics by ImpG^15^ and combined the imputed data with stage 2 summary statistics (Supplementary Note 1).

GERA: There were 6905 cases and 46,983 controls in GERA, and the sample prevalence was 12.4%. We cleaned the GERA genotype data using standard quality control (QC) filters (excluding SNPs with missing rate ≥ 0.02, Hardy-Weinberg equilibrium test *P* value ≤ 1 × 10^–6^ or minor allele count ≤ 1 and removing individuals with missing rate ≥ 0.02) and imputed the genotype data to the 1000 Genomes Projects (1KGP) reference panels^14^ using IMPUTE2^66^. We used GCTA^67^ (see URLs section) to compute the genetic relationship matrix (GRM) of all the individuals based on a subset of imputed SNPs (HapMap3 SNPs with MAF ≥ 0.01 and imputation info score ≥ 0.3), removed the related individuals at a genetic relatedness threshold of 0.05, and retained 53,888 individuals (6905 cases and 46,983 controls) for further analysis. We computed the first 20 principal components (PCs) from the GRM. The summary statistics in GERA were obtained from a GWAS analysis using PLINK2^31^ with sex, age, and the first 20 PCs fitted as covariates. To examine the influence of imputation panel on the meta-analysis result, we further imputed GERA to the HRC^68^ using the Sanger imputation service (see URLs section).

UKB: Genotype data from UKB were cleaned and imputed to HRC by the UKB team^13^. There were 21,147 cases and 434,460 controls, and the sample prevalence was 5.5%. We identified a European subset of UKB participants (*n* = 456,426) by projecting the UKB participants onto the 1KGP PCs. Genotype probabilities were converted to hard-call genotypes using PLINK2^31^ (hard-call 0.1), and we excluded SNPs with minor allele count < 5, Hardy-Weinberg equilibrium test *P* value < 1 × 10^–6^, missing genotype rate > 0.05, or imputation info score < 0.3. The UKB phenotype was acquired from self-report, ICD10 main diagnoses and ICD10 secondary diagnoses (field IDs: 20002, 41202, and 41204). The GWAS analysis in UKB was conducted in BOLT-LMM^30^ with sex and age fitted as covariates. In the BOLT-LMM analysis, we used 711,933 SNPs acquired by LD pruning (*r*^2^ < 0.9) from Hapmap3 SNPs to control for relatedness, population stratification and polygenic effects. We transformed the effect size from BOLT-LMM on the observed 0–1 scale to the OR using LMOR^69^.

### Inverse variance based meta-analysis

Before conducting the meta-analysis, we performed several analyses in which we examined genetic heterogeneity and sample overlap among data sets (Supplementary Note 2). We performed a 2-stage meta-analysis. The first stage combined DIAGRAM stage 1 (GWAS chip) data with GERA and UKB. The second stage combined DIAGRAM stage 1 and 2 (GWAS chip and metabolism chip) with GERA and UKB. We extracted the SNPs common to the 3 data sets (5,526,193 SNPs in stage 1 and 5,053,015 million SNPs in stage 2) and performed the meta-analyses using an inverse-variance based method in METAL^16^. The stage 2 meta-analysis data were used in the follow-up analyses.

### Summary-data-based Mendelian randomization analysis

We performed SMR and HEIDI analyses^10^ to identify genes whose expression levels were associated with a trait due to pleiotropy using summary statistics from GWAS and eQTL/mQTL studies. We first performed the SMR analysis to test for association between the expression level of each gene and the disease using the top associated cis-eQTL of the gene as an instrumental variable (in a Mendelian randomization analysis framework). There are at least two models consistent with an observed SMR association, i.e., pleiotropy (a genetic variant having effects on both trait and gene expression) and linkage (2 genetic variants in LD, one affecting the trait and another affecting gene expression). The HEIDI test^10^ uses multiple SNPs in a cis-eQTL region to distinguish pleiotropy from linkage by testing whether there is heterogeneity in SMR effects estimated at different SNPs in LD with the top associated cis-eQTL. We used the SMR and HEIDI methods to test for pleiotropic associations between gene expression and T2D, between DNAm and T2D, and between T2D-associated gene expression and T2D-associted DNAm. In the SMR analysis, we used eQTL summary data from the eQTLGen Consortium (*n* = 14,115 in whole blood), the CAGE (*n* = 2765 in peripheral blood)^34^ and the GTEx v7 release (*n* = 385 in adipose subcutaneous tissue, *n* = 313 in adipose visceral omentum, *n* = 153 in liver, *n* = 220 in pancreas and *n* = 369 from whole blood)^36^. In CAGE and eQTLGen, gene expression levels were measured using Illumina gene expression arrays; in GTEx, gene expression levels were measured by RNA-seq. The SNP genotypes in all cohorts were imputed to 1KGP. The cis-eQTL within 2 Mb of the gene expression probes with *P*_eQTL_ < 5 × 10^−8^ were selected as the instrumental variables in the SMR test. The mQTL summary data were obtained from genetic analyses of DNA methylation measured on Illumina HumanMethylation450 arrays (*n* = 1980 in peripheral blood)^35^. We used mQTL data generated by the 450 K methylation arrays rather than whole-genome bisulfite sequencing (WGBS) because WGBS-based mQTL data of large sample size (at least 100 s) are not available yet. We demonstrated the statistical power of SMR test in our study by simulation under a pleiotropy model (Supplementary Note 9 and Supplementary Fig. 10).

### Estimating the genetic architecture for T2D

The MAF- and LD-stratified GREML (GREML-LDMS) is a method for estimating SNP-based heritability that is robust to model misspecification^51,70^. For ease of computation, we limited the analysis to a subset of unrelated UKB individuals (15,767 cases and 104,233 controls); in this subset, we kept all 15,767 cases among the unrelated individuals to maximize the sample size of cases and randomly selected 104,233 individuals from 332,813 unrelated controls. We first estimated the segment-based LD score, stratified ~18 million SNPs into 2 groups based on the segment-based LD scores (high vs. low LD groups separated by the median), and then stratified the SNPs in each LD group into 7 MAF bins (10^−4^ to 10^−3^, 10^−3^ to 10^−2^, 10^−2^ to 10^−1^, 0.1–0.2, 0.2–0.3, 0.3–0.4, and 0.4–0.5). We computed the GRMs using the stratified SNPs and performed GREML analysis fitting 14 GRMs (with sex, age, and the first 10 PCs fitted as covariates) in one model to estimate the SNP-based heritability in each MAF bin. We used 10% as the population prevalence to convert the estimate to that on the liability scale.

We used GCTB-BayesS^54^ to estimate the joint distribution of SNP effect size and allele frequency. This analysis is based on 348,580 unrelated individuals (15,767 cases and 332,813 controls) and HapMap3 SNPs (~1.23 million) with sex, age, and the first 10 PCs fitted as covariates. Each SNP effect has a mixture prior of a normal distribution and a point mass at zero, with an unknown mixing probability, *π*, representing the degree of polygenicity. The variance in effect size is modeled to be dependent on MAF through a parameter *S*. Under an evolutionarily neutral model, SNP effect sizes are independent of MAF, i.e., *S* = 0. A negative (positive) value of *S* indicates that variants with lower MAF are prone to having larger (smaller) effects, consistent with a model of negative (positive) selection. A Markov-chain Monte Carlo (MCMC) algorithm was used to draw posterior samples for statistical inference. The posterior mean was used as the point estimate, and the posterior standard error was approximated by the standard deviation of the MCMC samples. We conducted the analysis chromosome-wise for ease of computation.

### URLs

For MAGIC consortium, see https://www.magicinvestigators.org/. For DrugBank, see https://www.drugbank.ca/. For DrugBank documentation, see https://www.drugbank.ca/documentation. For GWAS catalog, see http://www.ebi.ac.uk/gwas/. For DIAGRAM summary data, see http://www.diagram-consortium.org/. For Sanger imputation service, see https://imputation.sanger.ac.uk/. For GCTA, see http://cnsgenomics.com/software/gcta/. For GCTB, see http://cnsgenomics.com/software/gctb/.

### Data availability

Summary statistics from the meta-analysis are available at http://cnsgenomics.com/data.html.

## Electronic supplementary material

Supplementary Information

Description of Additional Supplementary Files

Supplementary Data 1

Supplementary Data 2

Supplementary Data 3

Supplementary Data 4

Supplementary Data 5

Supplementary Data 6

Supplementary Data 7

Supplementary Data 8

Supplementary Data 9

Supplementary Data 10

Supplementary Data 11

Supplementary Data 12

Supplementary Data 13

Supplementary Data 14

Supplementary Data 15
