# Supplementary material for: Genome-wide association analyses identify 143 risk variants and putative regulatory mechanisms for type 2 diabetes
Source: Nat Commun. 2018 Jul 27;9:2941. doi: 10.1038/s41467-018-04951-w (PMC6063971; doi:10.1038/s41467-018-04951-w)
Supplement: Supplementary file 1 — Supplementary Information [file 41467_2018_4951_MOESM1_ESM.pdf]

Genome-wide association analyses identify 143 risk variants and putative regulatory mechanisms for type 2 diabetes

Xue *et al.*

Supplementary Note 1 Imputing the stage 1 data of DIAGRAM to 1KGP using ImpG

Since individual-level genotypes are not available in DIAGRAM, we imputed the stage 1 summary statistics of DIAGRAM to 1KGP using ImpG[1]. Before imputation, we removed SNPs on less than 9,000 cases or 50,000 controls. The haplotype reference panel files (EUR) and SNP mapping files were obtained from 1KGP phase 1 (release v3). We chose phase 1 because the ImpG-sum software does not take INDELs (insertions and deletions) into account. After removing SNPs with MAF < 0.01 or imputation accuracy metric[1] $r^2_{pred} < 0.8$, 6,233,351 SNPs were retained for further analysis.

Supplementary Note 2 Heterogeneity and sample overlap among data sets

Before meta-analysis, we applied the LD score regression approach[2,3] to estimate the genetic correlation and sample overlap between pairwise data sets, and to assess the inflation in test-statistics in each data set. The estimates of genetic correlation between pairwise data sets were all not significantly different from 1 (**Supplementary Table 1**), suggesting the lack of evidence for genetic heterogeneity. The estimates of bivariate LD score regression intercept between pairwise data sets were all close to zero, suggesting the lack of evidence for sample overlap. We also used a metric called λ_{meta}[4] to test for sample overlap between pairwise data sets ($\lambda_{meta} > 1$ if there is sample overlap between two data sets). λ_{meta} was 1.0 between DIAGRAM and GERA, 1.0 between DIAGRAM and UKB, and 0.99 between GERA and UKB, again suggesting the lack of evidence for sample overlap among the three data sets.

The estimate of intercept of the univariate LD score regression was 1.009 (*s.e.* = 0.008) in DIAGRAM, 1.031 (*s.e.* = 0.009) in GERA, and 1.057 (*s.e.* = 0.013) in UKB, suggesting that population stratification has been well controlled although the small inflation in GREA and UKB is worth further investigation. We performed a BOLT-LMM[5] analysis in GERA and re-ran the univariate LD score regression using the GWAS summary statistics from BOLT-LMM. The intercept was 1.026 (*s.e.* = 0.008), similar to the estimate above and still significantly larger than 1, suggesting that the small inflation in the LD score regression intercept in GERA is unlikely to be due to population structure. Note that a small inflation in LDSC intercept is often observed in data with large sample sizes[6]. Nevertheless, the remaining inflation in LDSC intercept from the BOLT-LMM summary data might be due to inflated test-statistics from a mixed linear model analysis of unbalanced case-control ratio[7].

Supplementary Note 3 Functional relevance of the novel gene loci to T2D

The functional relevance of some novel gene loci to T2D are supported by existing biological or molecular evidence related to insulin and glucose. For example, *MBNL1* was up-regulated by

insulin stimulation[8] and controlled insulin receptor (*INSR*) exon inclusion by binding to a downstream enhancer[9]. A missense mutation in *STAT3* was reported to result in neonatal diabetes by reducing insulin synthesis[10] or premature induction of pancreatic differentiation[11]. A neighbouring gene of *STAT3* (~14kb distance), *PTRF*, was reported to be associated with glucose tolerance status[12] and mediate insulin-regulated gene expression[13]. Activation of *CAMK2G* suppressed hepatic insulin signalling (insulin resistance)[14] and *FOXA2* could improve the hepatic insulin resistance in diabetic/insulin-resistant mice models[15].

Supplementary Note 4 GCTA-fastBAT analysis

Because the effect size of an individual SNP is often very small, it would be more powerful to detect the aggregated effect of a set of SNPs at the locus that harbours multiple associated variants. fastBAT[16] is a set-based association test approach using summary data from GWAS to test the aggregated effect of a set of SNPs within a gene[16]. We applied fastBAT to run a gene-based test using the summary-level data from the meta-analysis with LD between SNPs estimated from the 1KGP-imputed GERA data. We clustered the SNPs into 24,765 genes by physical distance and tested each gene for association at a genome-wide significance level ($P < 0.05/24{,}765 = 2.02 \times 10^{-6}$). Here, we define a novel gene discovery as a gene that passed genome-wide significance level ($P_{\text{fastBAT}} < 2.02 \times 10^{-6}$) in the gene-based analysis but there is no genome-wide significant SNP ($P_{\text{GWAS}} > 5 \times 10^{-8}$) within ± 0.5 Mb of the gene.

We identified 374 genes (12 novel genes in addition to the single-SNP based meta-analysis) (**Supplementary Data 2**) for T2D at $P < 2.0 \times 10^{-6}$. The gain of power in the gene-based test can be due to the reduced multiple-testing burden or multiple independently associated variants at a locus. We therefore performed a conditional analysis at each of the 12 loci, and found that there were multiple independent signals with $P < 5 \times 10^{-5}$ at 4 of these loci (*NDUFS3*, *HIVEP2*, *ITGA1*, *FAM110D*) (**Supplementary Fig. 5**).

Supplementary Note 5 GCTA-COJO analysis

Conditional and joint analysis[17] (COJO) aims to identify multiple signals in a locus, conditioning on the primary associated SNP. In COJO analysis, we performed a stepwise model selection procedure to select near-independent SNPs. We set the threshold *P*-value to 5×10^{-8}, and window size of 10Mb, assuming that SNPs more than 10Mb away from each other or on different chromosomes are in linkage equilibrium. We used 1KGP-imputed GERA dataset as the reference for LD estimation. We identified 139 SNPs at the genome-wide significance threshold (**Supplementary Data 3**). There were seven loci with multiple independent signals associated with T2D (**Supplementary Data 3**). The joint effects of the SNPs at the seven loci estimated from

GCTA-COJO using summary-level data were consistent with those from multiple regression analysis of individual-level data from UKB (**Supplementary Data 4**).

Supplementary Note 6 Polygenic risk score (PRS) analysis

We used DIAGRAM and UKB as the discovery set and GERA as a validation set in the PRS analysis[18]. To avoid sample-overlap between the discovery and validation sets, we re-ran the meta-analysis excluding the GERA cohort and identified 109 near-independent common SNPs at $P < 5\times10^{-8}$ by clumping (LD r^2 threshold = 0.01 and window size = 1 Mb). These SNPs were then used to derive prediction equations for individuals in GERA. We divided GERA into ten subsets (each with sample size ~6,000 and similar sample prevalence) to acquire the sampling variance of the estimated classification accuracy. On average, the classification accuracy (measured by the area under the curve or AUC[19]) was 0.579 (*s.e.* = 0.003), lower than the classification accuracy of 0.599 (*s.e.* = 0.002) obtained using all SNP effects (~5.1 million SNPs) estimated from GCTA-SBLUP (Summary-data-based Best Linear Unbiased Prediction)[20] (**Supplementary Table 2**). We further quantified the proportion of variance in liability to T2D in GERA explained by a polygenic predictor computed from the 109 genome-wide significant SNPs discovered in the meta-analysis of DIAGRAM and UKB. The polygenic predictor explained 2.1% of the variance in liability to T2D, about a third of the estimate of h^2_{SNP} on the liability scale (note that the $\hat{h}^2_{\mathrm{SNP}}$ in GERA was much lower than that in UKB).

Supplementary Note 7 Enrichment of genetic variation in functional regions and tissue/cell types

Recent studies have indicated that different functional regions of the genome contribute disproportionately to total heritability[21]. We applied a stratified LD score regression method[21] to dissect the contributions of the functional elements to the SNP-based heritability ($\hat{h}^2_{\mathrm{SNP}}$) for T2D. There were significant enrichments in some functional categories (**Supplementary Fig. 6** and **Supplementary Data 5**). First, the conserved regions in mammals[22] showed the largest enrichment, with 2.6% of SNPs explaining 24.8% of $\hat{h}^2_{\mathrm{SNP}}$ (fold-change = 9.5; $P = 1.9\times10^{-4}$). This supports the biological importance of conserved regions, although the functions of many conserved regions are still undefined. Second, the histone marker H3K9ac[23] was highly enriched, with 12.6% of SNPs explaining 59.7% of $\hat{h}^2_{\mathrm{SNP}}$ (fold-change = 4.7; $P = 2.5\times10^{-5}$). H3K9ac can activate genes by acetylation and is highly associated with active promoters. We also partitioned $\hat{h}^2_{\mathrm{SNP}}$ into ten cell type groups (**Supplementary Table 3**); the top cell type group for T2D was "adrenal or pancreas" (fold-change = 6.0; $P = 8.1\times10^{-9}$), and the result was highly significant ($P_{\mathrm{Bonferroni}} = 1.8\times10^{-6}$) after Bonferroni correction for 220 tests (**Supplementary Fig. 7**).

We further used MAGMA[24] to test the enriched gene sets. In total, 305 gene sets in GO_BP terms and 20 gene sets in KEGG pathways were significantly enriched (**Supplementary Data 6**). The top pathway enrichment was "glucose homeostasis" (P = 6.0×10^{-8}) in GO_BP, and "maturity onset diabetes of the young" (P = 3.2×10^{-7}) in KEGG. To further investigate the molecular connections of T2D-associated genes, a protein-protein interaction network was analyzed using STRING[25] (**Supplementary Fig. 8**). Among the functional enrichment (**Supplementary Data 7**) in this network, there are four genes (*HHEX*, *HNF1A*, *HNF1B*, and *FOXA2*) involved in the KEGG pathway of "maturity onset diabetes of the young", and four genes (*ADCY5*, *CAMK2G*, *KCNJ11*, and *KCNU1*) were enriched in "insulin secretion".

Supplementary Note 8 Enrichment of the T2D-associated DNA methylation sites in functional categories

We obtained chromatin status data of 127 epigenomes from the Roadmap Epigenomics Mapping Consortium[26]. We mapped 235 T2D-associated DNA methylation (DNAm) sites to the 14 functional categories defined in Wu *et al.*[27] and counted the number of DNAm sites mapped to each category. We then randomly sampled from all the DNAm probes the same number of null probes with variance in DNAm levels at each probe matched and repeated the sampling 500 times. The fold enrichment value was calculated as a ratio of the observed value to that of a null probe set averaged across 500 replicates. The standard error of estimate of fold enrichment was calculated from 500 replicates (**Supplementary Fig. 9**).

Supplementary Note 9 SMR power calculation

To illustrate the power of the SMR test as a function of the sample size of eQTL study, we performed simulations under a pleiotropy model (i.e. mimicking a shared causal variant between the expression level of a gene and the disease). Following Zhu et al.[28], we simulated the causal SNP from a binomial distribution $z \sim Binomial(2, f)$ with f being the minor allele frequency (MAF), $f \sim Uniform(0.01, 0.5)$. Gene expression level (x) was simulated based on the model $x = zb_{\mathrm{zx}} + e_{\mathrm{zx}}$, where $e_{\mathrm{zx}} \sim N(0, var(zb_{\mathrm{zx}})\ (1/R^2_{\mathrm{zx}} - 1))$ with R^2_{zx} being the proportion of variance in x explained by the causal SNP. We randomly sampled R^2_{zx} from the observed proportions of variance in gene expression levels explained by the corresponding top cis-eQTLs with $P_{\mathrm{eQTL}} < 5{\times}10^{-8}$ for the 27 significant genes in the eQTLGen data. The trait phenotype (y) was simulated based on the model $y = zb_{\mathrm{zy}} + e_{\mathrm{zy}}$, where $e_{\mathrm{zy}} \sim N\left(0, var\left(zb_{\mathrm{zy}}\right)(1/R^2_{\mathrm{zy}} - 1)\right)$ with R^2_{zy} being the proportion of variance in y explained by the causal SNP. We randomly sampled R^2_{zy} from the observed proportion of variance in T2D risk explained by the top associated SNPs (on the liability scale) at the genome-wide significant loci (i.e. $P_{\mathrm{GWAS}} <$

$5{\times}10^{-8}$) identified in our T2D meta-analysis. The variance explained in the meta-analysis data was computed using an approximate approach assuming that the effect size of an individual SNP is small[18], i.e. $R^2 = 2f(1-f)(OR-1)^2/i^2$, where $i = v/K$ with K being the disease prevalence and v the height of the normal curve at the truncation point pertaining to K. We set $n_{\mathrm{GWAS}} = 659{,}316$ to be consistent with the sample size of our T2D meta-analysis and varied the sample size of eQTL study (n_{eQTL}) from 200 to 2000. We dichotomized y to a 0-1 trait assuming a disease prevalence of 10%. This simulation was repeated 5000 times.

The overall power to detect the simulated pleiotropic association between gene and trait depends on the power of detecting the eQTL effect (because the SMR test is performed only for genes with $P_{\mathrm{eQTL}} < 5{\times}10^{-8}$) and the power of the SMR test (the SMR test-statistic is a monotonic function of n_{eQTL})[28]. The simulation result showed that SMR power for either discovery or replication was high even using eQTL data with relatively small sample size (e.g. GTEx data), but the overall power was restricted by the power of eQTL detection (**Supplementary Fig. 10**). In practice, the power is likely to be slightly lower than that quantified by this simulation because we used the estimated variance explained, which are biased upwards due to the winner's curse, as the true parameters for simulation.

Supplementary Note 10 Potential adverse effects and additional drug targets for SMR hits

To explore whether any of these three genes have potential adverse effects, we checked the associations of the lead variants at the three loci with other traits from previous studies, including two insulin-related GWAS (insulin sensitivity[29] and insulin secretion[30]) and four lipid traits (HDL cholesterol, LDL cholesterol, triglycerides and total cholesterol)[31] (**Supplementary Data 14**). We did not observe any significant association with insulin traits after correcting for multiple testing (*i.e.*, 0.05 / ($3{\times}t$), where t is the number of traits). However, the risk allele of the lead T2D-associated variant at the *LTA* locus was associated with increased LDL cholesterol, total cholesterol and triglycerides. The risk allele of the lead T2D-associated variant at the *ARG1* locus was associated with decreased HDL cholesterol and total cholesterol.

In addition to the three genes that are currently targeted by approved drugs, we found two additional genes that are targeted by an approved veterinary drug and a nutraceutical drug (See **URLs** for detailed definition), respectively. *PLEKHA1* (UniProt ID: Q9HB21), whose expression level was negatively associated with T2D risk, is targeted by citric acid (DrugBank ID: DB04272). Intraperitoneal injection of citrate in diabetic mice reduced apoptotic and inflammatory responses and protected cardiac abnormalities induced by diabetes[32]. A reduction of citric acid cycle (CAC) flux which reflects mitochondrial dysfunction was observed in T2D patients[33].

EHHADH (UniProt ID: Q08426), whose expression level was negatively associated with T2D risk, is targeted by a nutraceutical drug NADH (DrugBank ID: DB00157).

Supplementary Note 11 Sex or age heterogeneity analysis

We performed a GWAS analysis within each sex (male or female) or age (two age categories separated at median year of birth) group in the UKB data. In the sex heterogeneity analysis, there were 208,419 males and 247,188 females. In age heterogeneity analysis, there were 218,261 individuals in the first age group (born from 1951 to 1971) and 237,346 individuals in the second age group (born from 1930 to 1950). We then tested the difference in the estimated SNP effects between the two sex (or age) groups by a heterogeneity test, i.e. $T_d = (\hat{b}_1 - \hat{b}_2)^2 \,/\, (SE_1^2 + SE_2^2)$, which follows a χ^2 distribution with $df = 1$ under the null hypothesis of no difference.

There was no evidence for sex heterogeneity (**Supplementary Fig. 14**), consistent with the observation that the male-female genetic correlation estimated by bivariate LDSC[3] was not significantly different from 1 ($\hat{r}_g = 0.94$, $s.e. = 0.042$, and $P_{\text{difference}} = 0.17$). There was only one genome-wide significant signal (rs72805579 at the *TMEM17* locus with $P_{\text{heterogeneity}} = 2.1\times10^{-9}$) with age heterogeneity (**Supplementary Fig. 14**). The estimates of SNP effects were of opposite directions in the two age groups, but the effect was not genome-wide significant in either age group (**Supplementary Table 8**).

Supplementary Note 12 Biases in SNP-T2D associations due to misdiagnosed T1D or LADA cases

Most data used in this study were from DIAGRAM and UKB. The summary statistics of DIAGRAM were from a meta-analysis of 12 GWAS cohorts. Previous studies show that the biases in SNP-T2D associations due to misdiagnosis are likely to be very modest[34-36]. Those studies claimed that their GWAS results for T2D were not confounded by T1D associations because of the absence of most known T1D-associated loci in their T2D discovery. Following the approach of Mahajan *et al.*[35], we extracted 48 T1D-associated SNPs from Bradfield *et al.*[37], and found that only four (16q23.1, *GLIS3*, 6q22.32 and *PTPN22*) of them showed associations with T2D at a suggestive significance level (i.e. $P < 1\times10^{-5}$) with two of them (*GLIS3* and 6q22.32) passing the genome-wide significance level (i.e. $P < 5\times10^{-8}$) (**Supplementary Data 15**).

Furthermore, following Scott *et al.*[36], we computed the polygenic risk score (PRS) for T1D in UKB using the 48 T1D-associated SNPs and tested for its association with the T2D phenotype. The PRS computed from the 44 T1D-only SNPs did not show significant association with T2D (OR=1.02 and $P = 0.93$), unless the four risk loci that showed suggestive associations with T2D discovery were included in the computation of PRS (Odds ratio (OR) =1.99 and $P = 5.86\times10^{-13}$). These results

suggest that our samples were unlikely to include a substantial number of misdiagnosed T1D cases, and that the associations of the four T1D loci with T2D were likely because of pleiotropy. We further performed the T2D GWAS by logistic regression using individual-level data with and without fitting the T1D phenotype as covariate in unrelated UKB individuals (n = 347,997). The correlation coefficient of z-statistics of the 139 significant loci between the conditional and unconditional models was almost one (Pearson correlation r = 0.995). In the unconditional model, 43 out of 139 loci remained genome-wide significant at $P < 5 \times 10^{-8}$ in this subset. Among these 43 loci, 34 were still significant after conditional on T1D phenotype. For those loci that were not significant conditioning on the T1D phenotype, the differences in P-value were mostly marginal except for two SNPs in MHC region: rs1063355 located in *HLA-DQB1*, $P_{\text{unconditional}} = 1.1 \times 10^{-15}$, $P_{\text{conditional}} = 1.3 \times 10^{-4}$; rs2071479 located in *HLA-DOB*, $P_{\text{unconditional}} = 5.2 \times 10^{-7}$, $P_{\text{conditional}} = 5.8 \times 10^{-1}$, indicating that most T2D loci detected in this study were independent of T1D.

We could not find any publicly available data for LADA to perform the analyses above. However, a recent study[38] commented that "type 1 diabetes genetic risk score provides a reasonable approximation for all autoimmune diabetes". Under this hypothesis, our conclusion on T1D misdiagnosis is expected to hold for LADA. In addition, previous studies identified three T2D associations due to misdiagnosed LADA cases, i.e., *HLA-DQB1*[39,40], *INS*[39] and *PTPN22*[39]. Two of them (*INS* and *PTPN22*) were not genome-wide significant and *HLA-DQB1* was not a novel locus in our analysis.

In conclusion, the novel loci identified in this study were very unlikely due to misdiagnosed T1D or LADA cases.

Supplementary Note 13 Acknowledgements

UKB: This study has been conducted using UK Biobank resource under Application Number 12514. UK Biobank was established by the Wellcome Trust medical charity, Medical Research Council, Department of Health, Scottish Government and the Northwest Regional Development Agency. It has also had funding from the Welsh Assembly Government, British Heart Foundation and Diabetes UK.

GERA (dbGaP accession: phs000674.v2.p2)**:** The Genetic Epidemiology Research on Adult Health and Aging study was supported by grant RC2 AG036607 from the National Institutes of Health, grants from the Robert Wood Johnson Foundation, the Ellison Medical Foundation, the Wayne and Gladys Valley Foundation and Kaiser Permanente. The authors thank the Kaiser Permanente Medical Care Plan, Northern California Region (KPNC) members who have generously agreed to

participate in the Kaiser Permanente Research Program on Genes, Environment and Health (RPGEH).

We also thank DIAGRAM (DIAbetes Genetics Replication And Meta-analysis) consortium, MAGIC (the Meta-Analyses of Glucose and Insulin-related traits Consortium) and the Global Lipids Genetics Consortium (GLGC), for providing GWAS summary statistics for public access.

Supplementary Table 1 Results from bivariate LD score regression analysis (with standard errors) in three cohorts

Sample 1	Sample 2	Genetic correlation	Intercept
DIAGRAM	GERA	1.0659 (0.1165)	0.0120 (0.0069)
GEAR	UKB	1.0830 (0.0830)	0.0172 (0.0079)
DIAGRAM	UKB	0.9838 (0.0541)	0.0198 (0.0076)

Supplementary Table 2 The accuracy of using SNP effects estimated from UKB and DIAGRAM to classify T2D cases and controls in GERA

Methods		P-value < 5E-08	P-value < 1E-05	All SNPs
**Clumping**	AUC	0.5791	0.5979	/
	s.e.	0.0029	0.0030	/
**SBLUP**	AUC	0.5778	/	0.5999
	s.e.	0.0029	/	0.0022

Notes: AUC is calculated using R-package "pROC".

Supplementary Table 3 Enrichment of the variance explained by SNPs in 10 different cell type groups for T2D

Category	Prop._SNPs	Prop._h^2	Prop._h^2s.e.	Enrichment	Enrichment s.e.	Enrichment p-value	Coefficient	Coefficient s.e.	Coefficient z-score
Adrenal or pancreas	0.094	0.561	0.082	5.999	0.877	8.05E-09	3.83E-08	8.14E-09	4.708
CNS	0.149	0.395	0.078	2.653	0.523	1.23E-03	-3.91E-09	5.05E-09	-0.774
Cardiovascular	0.111	0.413	0.076	3.719	0.683	7.34E-05	5.99E-09	5.68E-09	1.054
Connective or Bone	0.115	0.427	0.092	3.711	0.803	5.99E-04	6.99E-09	8.03E-09	0.870
Gastrointestinal	0.168	0.543	0.112	3.236	0.670	8.97E-04	4.76E-09	6.35E-09	0.750
Immune or hematopoietic	0.233	0.652	0.089	2.792	0.383	4.17E-06	1.08E-08	4.95E-09	2.174
Kidney	0.043	0.204	0.049	4.797	1.139	8.45E-04	1.67E-08	9.62E-09	1.733
Liver	0.072	0.352	0.076	4.872	1.051	1.87E-04	2.23E-08	1.02E-08	2.185
Skeletal Muscle	0.104	0.338	0.077	3.254	0.742	2.44E-03	-4.14E-09	6.50E-09	-0.636
Other	0.203	0.754	0.146	3.717	0.718	2.10E-04	1.70E-08	8.43E-09	2.014

Prop._SNPs: proportion of SNPs. Prop._h^2: proportion of SNP-based h^2 explained.

Supplementary Table 4 Independent rare variants associated with T2D from the GWAS analysis in UKB at BOLT_LMM p-value < 5E-8

CHR	SNP	A1	A2	BOLT_LMM in the whole UKB (n = 455,607 including relatives)			PLINK2 in UKB (n = 348,580 unrelated individuals)			Mapped Genes
				**A1FREQ**	**OR (95% CI)**	***P***	**A1FREQ**	**OR (95% CI)**	***P***	
1	rs527320094	C	A	0.00023	2.77 (1.81-3.74)	4.60E-09	0.00023	3.48 (2.21-5.49)	8.20E-08	*LOC105378797*
2	rs184847416	T	C	0.00012	3.54 (1.95-5.12)	1.10E-08	0.00012	3.96 (2.19-7.16)	4.97E-06	*UBBP1*
5	rs146886108	T	C	0.00714	0.71 (0.63-0.79)	4.40E-09	0.00711	0.65 (0.56-0.77)	3.42E-07	*ANKH*
5	rs78408340	G	C	0.00960	1.34 (1.24-1.43)	4.40E-14	0.00959	1.43 (1.29-1.58)	4.02E-12	*PAM*
7	rs551513405	A	G	0.00016	3.05 (1.83-4.28)	2.40E-08	0.00016	2.90 (1.60-5.24)	4.45E-04	*GBAS*
9	rs79768058_T_G	G	T	0.00020	2.87 (1.81-3.93)	1.20E-08	0.00019	3.34 (2.03-5.50)	2.09E-06	*CFAP77*
10	rs117229942	T	C	0.00818	0.70 (0.62-0.77)	4.00E-11	0.00823	0.64 (0.55-0.75)	1.80E-08	*TCF7L2*
12	rs576083050	T	C	0.00035	2.34 (1.62-3.05)	3.00E-08	0.00034	2.32 (1.52-3.54)	9.55E-05	*CCDC77*
14	rs559651557	C	T	0.00023	2.67 (1.72-3.62)	3.30E-08	0.00023	3.47 (2.21-5.44)	6.59E-08	*SFTA3*
15	rs79890196	C	G	0.00026	2.60 (1.71-3.48)	2.40E-08	0.00025	3.22 (2.05-5.05)	3.57E-07	*NR2F2-AS1*
16	rs551640889	G	C	0.00012	3.61 (2.01-5.21)	5.00E-09	0.00013	4.15 (2.30-7.51)	2.38E-06	*XYLT1*

Notes: A1: minor allele; A2: major allele; A1FREQ: allele frequency of A1; SNPs with BOLT_LMM p-value < 5E-9 are highlighted in grey; PLINK2: we used a logistic regression with sex, age and first 10 PCs fitted as covariates.

Supplementary Table 5 Variance explained by SNPs in different MAF and LD bins estimated from the GREML-LDMS analysis in UKB

MAF bin	LD region	Estimate of variance explained	s.e.
1e-4~1e-3	low LD	0.0146	0.0152
	high LD	0.0000	0.0091
1e-3~1e-2	low LD	0.0215	0.0127
	high LD	0.0079	0.0073
0.01~0.1	low LD	0.0324	0.0076
	high LD	0.0269	0.0050
0.1~0.2	low LD	0.0389	0.0055
	high LD	0.0216	0.0038
0.2~0.3	low LD	0.0390	0.0054
	high LD	0.0164	0.0036
0.3~0.4	low LD	0.0312	0.0052
	high LD	0.0265	0.0038
0.4~0.5	low LD	0.0412	0.0047
	high LD	0.0142	0.0031
The sum of the estimates		0.3323	0.0211

Notes: The estimates based on the liability scale; The proportion of cases in the sample is 0.1314 and population disease prevalence is assumed to be 0.1.

Supplementary Table 6 Estimates of the relationship between MAF and effect size, proportion of SNPs with non-zero effects and SNP-based heritability from the BayesS analysis in UKB

CHR	S	*s.e.* of S	Pi	*s.e.* of Pi	h^2	*s.e.* of h^2	CHR_length (Mb)	Nr_SNPs
1	-1.0952	0.0347	0.0097	0.0026	0.0223	0.0022	249.25	110,148
2	0.0267	0.2748	0.0188	0.0077	0.0198	0.0019	243.20	111,127
3	-1.0165	0.1120	0.0073	0.0020	0.0215	0.0021	198.02	92,185
4	-0.1970	0.6380	0.0399	0.0316	0.0132	0.0019	191.15	82,209
5	-0.1935	0.2940	0.0860	0.0443	0.0126	0.0016	180.92	83,660
6	-1.0788	0.0356	0.0087	0.0022	0.0229	0.0021	171.12	86,843
7	-1.0735	0.0421	0.0077	0.0022	0.0162	0.0018	159.14	71,602
8	-0.3025	0.3329	0.0058	0.0023	0.0094	0.0016	146.36	71,953
9	-0.6040	0.3733	0.0043	0.0019	0.0092	0.0017	141.21	60,448
10	0.3629	0.4892	0.0011	0.0004	0.0145	0.0014	135.53	70,290
11	-1.0445	0.0463	0.0094	0.0025	0.0177	0.0018	135.01	67,512
12	-0.4090	0.3325	0.0128	0.0057	0.0115	0.0015	133.85	65,015
13	-1.1734	0.0444	0.0094	0.0033	0.0100	0.0015	115.17	49,298
14	1.2312	0.3306	0.1827	0.0670	0.0061	0.0011	107.35	43,261
15	-1.0550	0.0799	0.0130	0.0043	0.0108	0.0015	102.53	39,926
16	-0.0460	0.3955	0.0146	0.0058	0.0088	0.0012	90.35	41,931
17	0.0935	0.5217	0.0145	0.0062	0.0075	0.0012	81.20	35,701
18	-1.0728	0.1543	0.0118	0.0063	0.0065	0.0014	78.08	38,863
19	-0.9518	0.2204	0.0141	0.0054	0.0095	0.0014	59.13	24,061
20	-1.0833	0.0603	0.0079	0.0030	0.0079	0.0013	63.03	34,101
21	0.6059	0.8120	0.0136	0.0123	0.0011	0.0005	48.13	18,307
22	-0.7504	0.2775	0.0476	0.0270	0.0048	0.0011	51.30	18,668

Supplementary Table 7 Heritability on the liability scale estimated using different data sets and methods

Cohort	Methods	h^2_{SNP} of liability ($s.e.$)	Case	Control	Total	Prevalence	Nr. SNPs
Meta-analysis stage 1	LDSC	0.207 (0.011)	40,223	538,305	578,528	0.0695	1,031,721
Meta-analysis stage 2	LDSC	0.196 (0.011)	62,892	596,424	659,316	0.0954	1,006,749
UKB	GREML-LDMS	0.332 (0.021)	15,767	104,233	120,000	0.1314	18,138,214
UKB	BayesS	0.319 (0.041)	15,767	332,813	348,580	0.0452	1,317,109
UKB	HE-regression	0.287 (0.011)	15,767	332,813	348,580	0.0452	1,317,109
UKB	LDSC	0.226 (0.012)	21,147	434,460	455,607	0.0464	1,006,750

Note: We assume a population prevalence of 0.1 for T2D to convert the estimate of SNP-based h^2 on the observed scale to that on the liability scale.

Supplementary Table 8 Age heterogeneity

SNP	CHR	BP	A1	A2	Age group	A1 Freq.	*b*	*s.e.*	P_{GWAS}	*n*	$P_{heterogeneity}$
rs72805579	2	62719994	C	A	Born from 1930 to 1950	0.1218	-0.0808	0.0184	1.10E-05	237133	2.10E-09
					Born from 1951 to 1971	0.1236	0.1158	0.0271	2.00E-05	218071	

Notes: A1 Freq. = frequency of A1; b = estimated SNP effect with respect to A1; *n* = sample size.

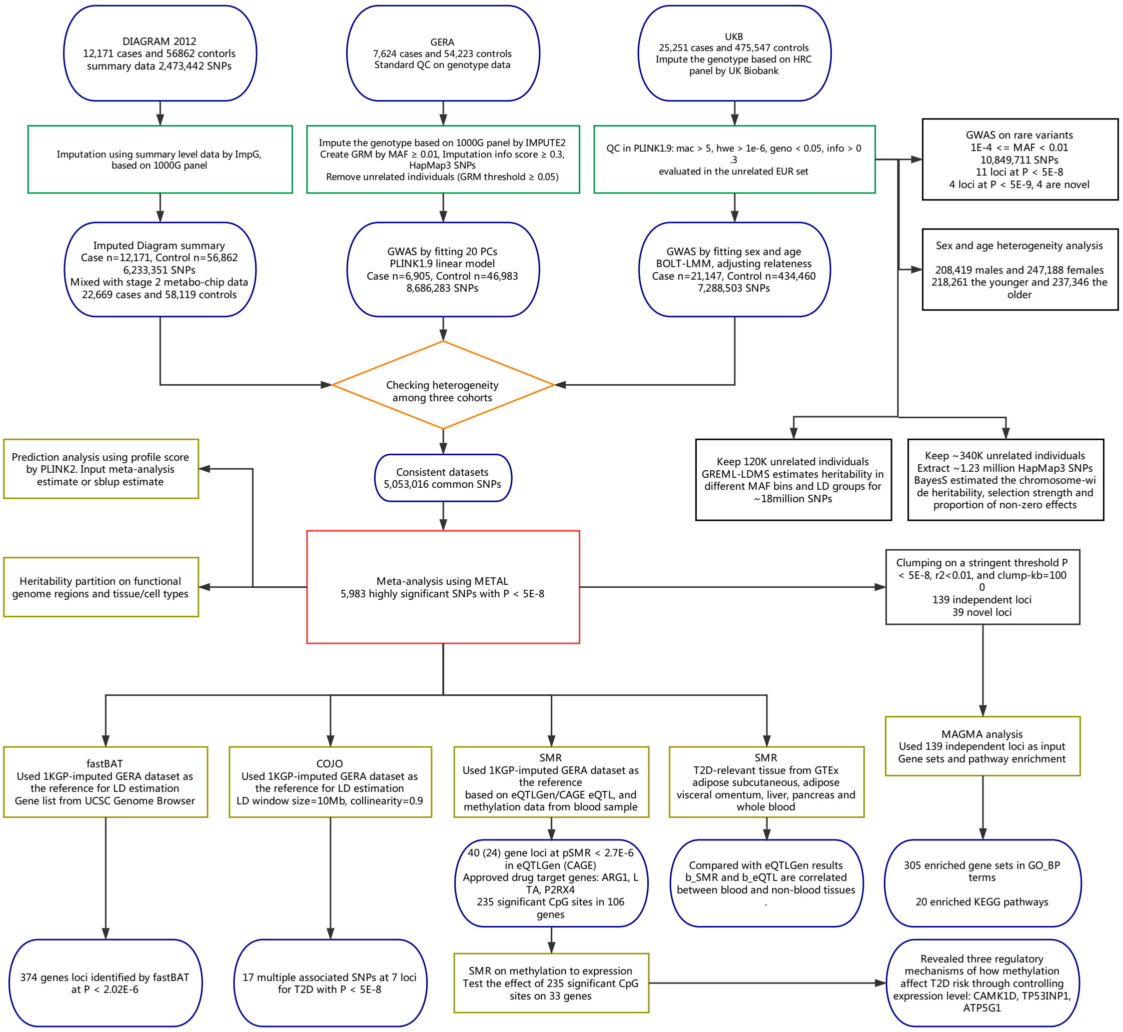

Supplementary Figure 1 Schematic diagram of this study.

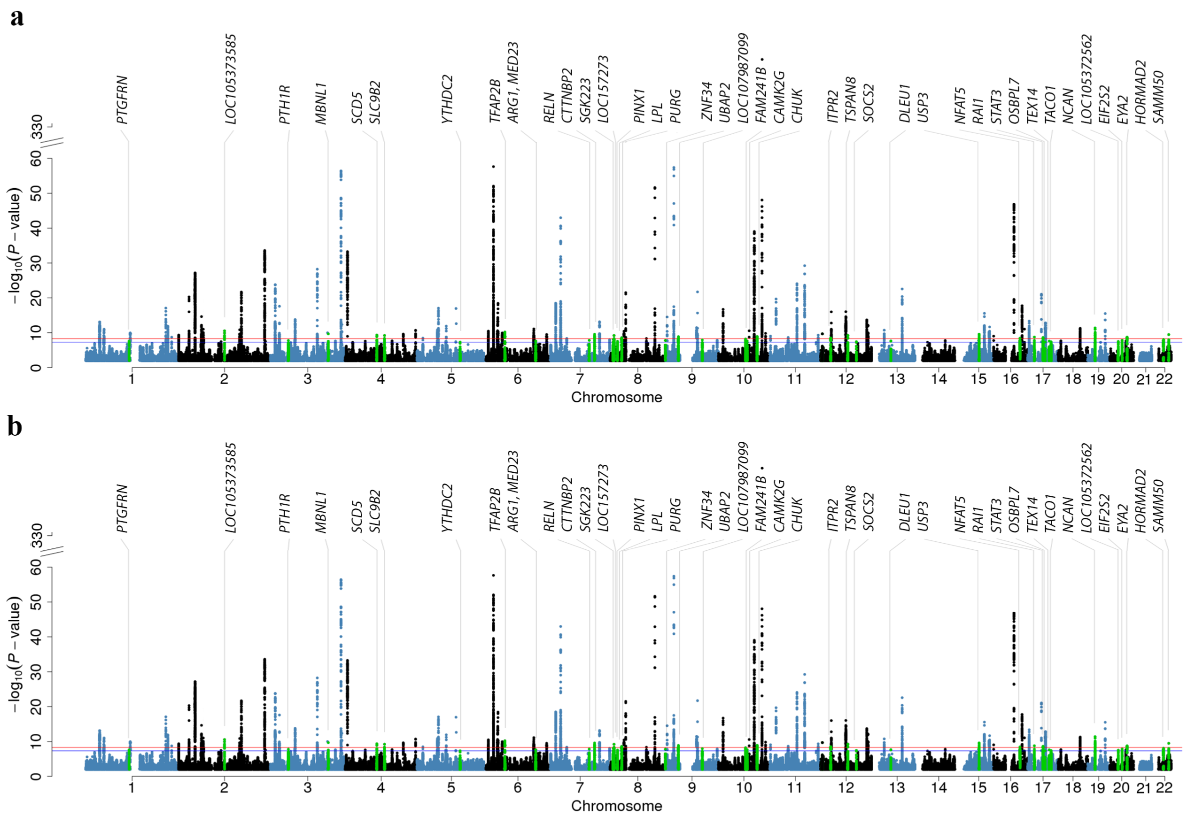

Supplementary Figure 2 Manhattan plots of meta-analysis with the GERA cohort imputed to different imputation reference panels. a) GERA was imputed to the 1000 Genomes Project (1000G). b) GERA was imputed to the Haplotype Reference Consortium (HRC) using the Sanger imputation server (https://imputation.sanger.ac.uk/). Shown are the associations for ~5 million common variants.

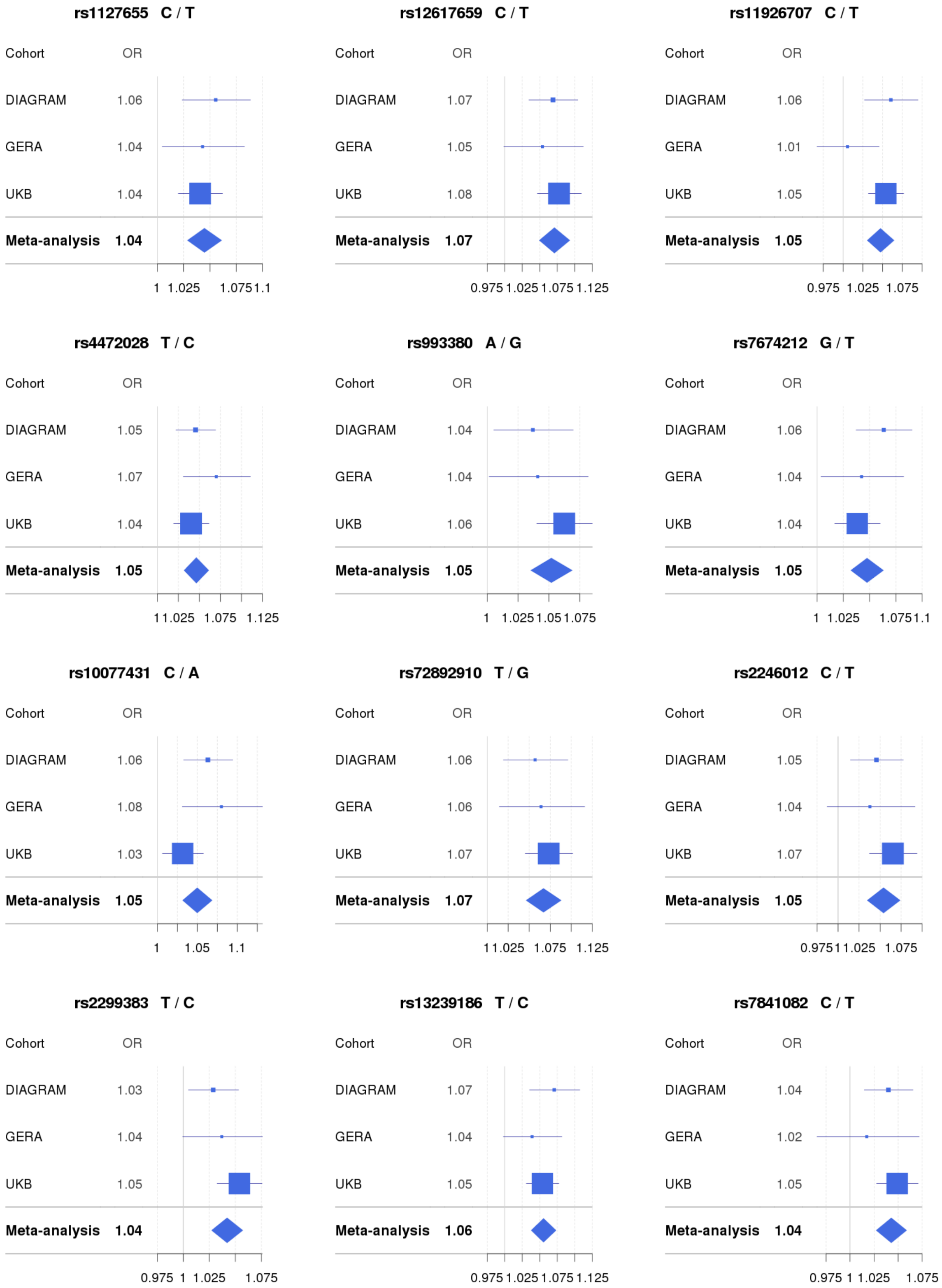

rs1127655 C / T
Cohort OR
DIAGRAM 1.06
GERA 1.04
UKB 1.04
Meta-analysis 1.04
1 1.025 1.075 1.1
rs12617659 C / T
Cohort OR
DIAGRAM 1.07
GERA 1.05
UKB 1.08
Meta-analysis 1.07
0.975 1.025 1.075 1.125
rs11926707 C / T
Cohort OR
DIAGRAM 1.06
GERA 1.01
UKB 1.05
Meta-analysis 1.05
0.975 1.025 1.075
rs4472028 T / C
Cohort OR
DIAGRAM 1.05
GERA 1.07
UKB 1.04
Meta-analysis 1.05
1 1.025 1.075 1.125
rs993380 A / G
Cohort OR
DIAGRAM 1.04
GERA 1.04
UKB 1.06
Meta-analysis 1.05
1 1.025 1.05 1.075
rs7674212 G / T
Cohort OR
DIAGRAM 1.06
GERA 1.04
UKB 1.04
Meta-analysis 1.05
1 1.025 1.075 1.1
rs10077431 C / A
Cohort OR
DIAGRAM 1.06
GERA 1.08
UKB 1.03
Meta-analysis 1.05
1 1.05 1.1
rs72892910 T / G
Cohort OR
DIAGRAM 1.06
GERA 1.06
UKB 1.07
Meta-analysis 1.07
1 1.025 1.075 1.125
rs2246012 C / T
Cohort OR
DIAGRAM 1.05
GERA 1.04
UKB 1.07
Meta-analysis 1.05
0.975 1 1.025 1.075
rs2299383 T / C
Cohort OR
DIAGRAM 1.03
GERA 1.04
UKB 1.05
Meta-analysis 1.04
0.975 1 1.025 1.075
rs13239186 T / C
Cohort OR
DIAGRAM 1.07
GERA 1.04
UKB 1.05
Meta-analysis 1.06
0.975 1.025 1.075 1.125
rs7841082 C / T
Cohort OR
DIAGRAM 1.04
GERA 1.02
UKB 1.05
Meta-analysis 1.04
0.975 1 1.025 1.075

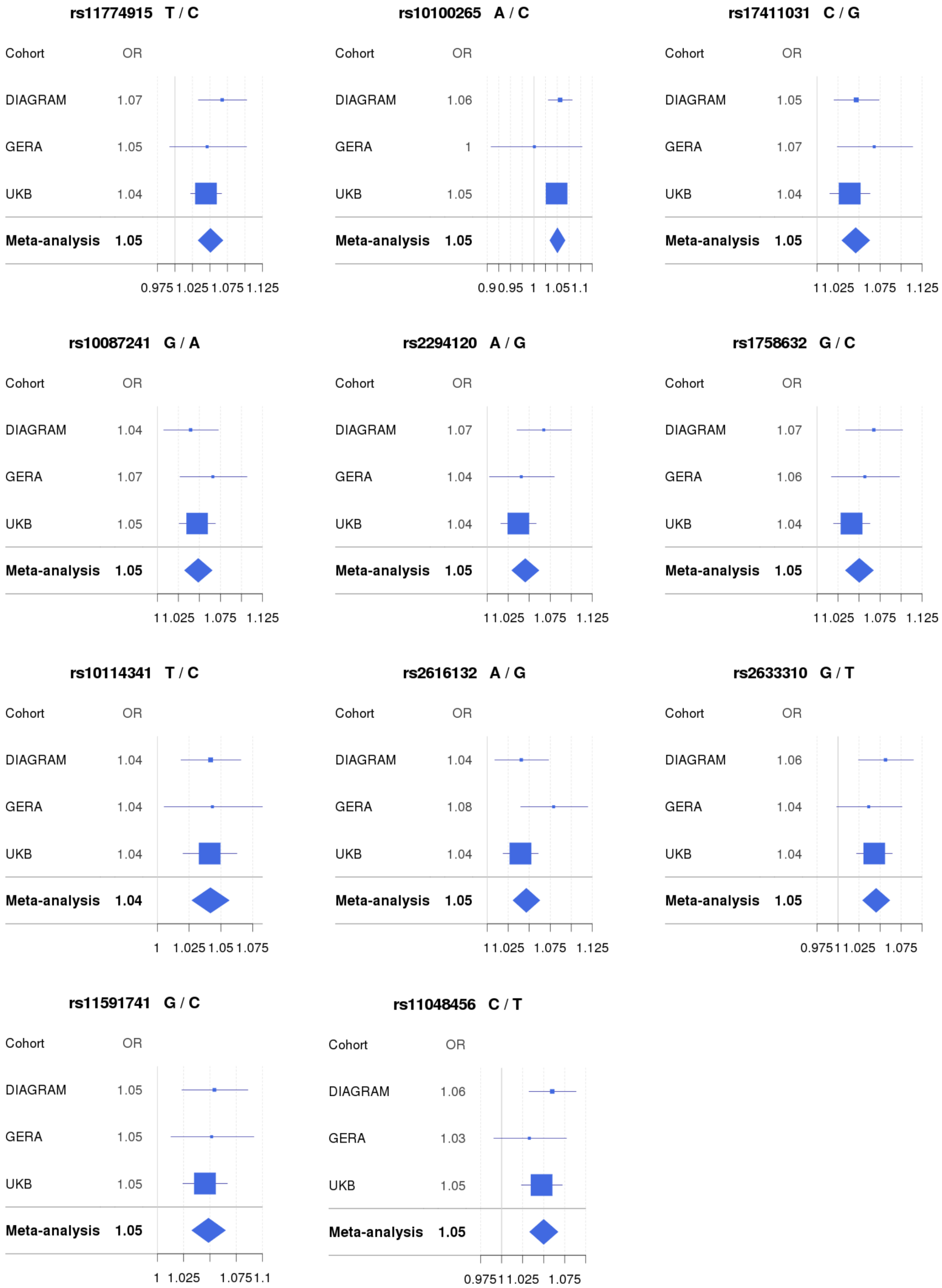
rs11774915 T / C
Cohort OR
DIAGRAM 1.07
GERA 1.05
UKB 1.04
Meta-analysis 1.05
0.975 1.025 1.075 1.125
rs10100265 A / C
Cohort OR
DIAGRAM 1.06
GERA 1
UKB 1.05
Meta-analysis 1.05
0.9 0.95 1 1.05 1.1
rs17411031 C / G
Cohort OR
DIAGRAM 1.05
GERA 1.07
UKB 1.04
Meta-analysis 1.05
1 1.025 1.075 1.125
rs10087241 G / A
Cohort OR
DIAGRAM 1.04
GERA 1.07
UKB 1.05
Meta-analysis 1.05
1 1.025 1.075 1.125
rs2294120 A / G
Cohort OR
DIAGRAM 1.07
GERA 1.04
UKB 1.04
Meta-analysis 1.05
1 1.025 1.075 1.125
rs1758632 G / C
Cohort OR
DIAGRAM 1.07
GERA 1.06
UKB 1.04
Meta-analysis 1.05
1 1.025 1.075 1.125
rs10114341 T / C
Cohort OR
DIAGRAM 1.04
GERA 1.04
UKB 1.04
Meta-analysis 1.04
1 1.025 1.05 1.075
rs2616132 A / G
Cohort OR
DIAGRAM 1.04
GERA 1.08
UKB 1.04
Meta-analysis 1.05
1 1.025 1.075 1.125
rs2633310 G / T
Cohort OR
DIAGRAM 1.06
GERA 1.04
UKB 1.04
Meta-analysis 1.05
0.975 1 1.025 1.075
rs11591741 G / C
Cohort OR
DIAGRAM 1.05
GERA 1.05
UKB 1.05
Meta-analysis 1.05
1 1.025 1.075 1.1
rs11048456 C / T
Cohort OR
DIAGRAM 1.06
GERA 1.03
UKB 1.05
Meta-analysis 1.05
0.975 1 1.025 1.075

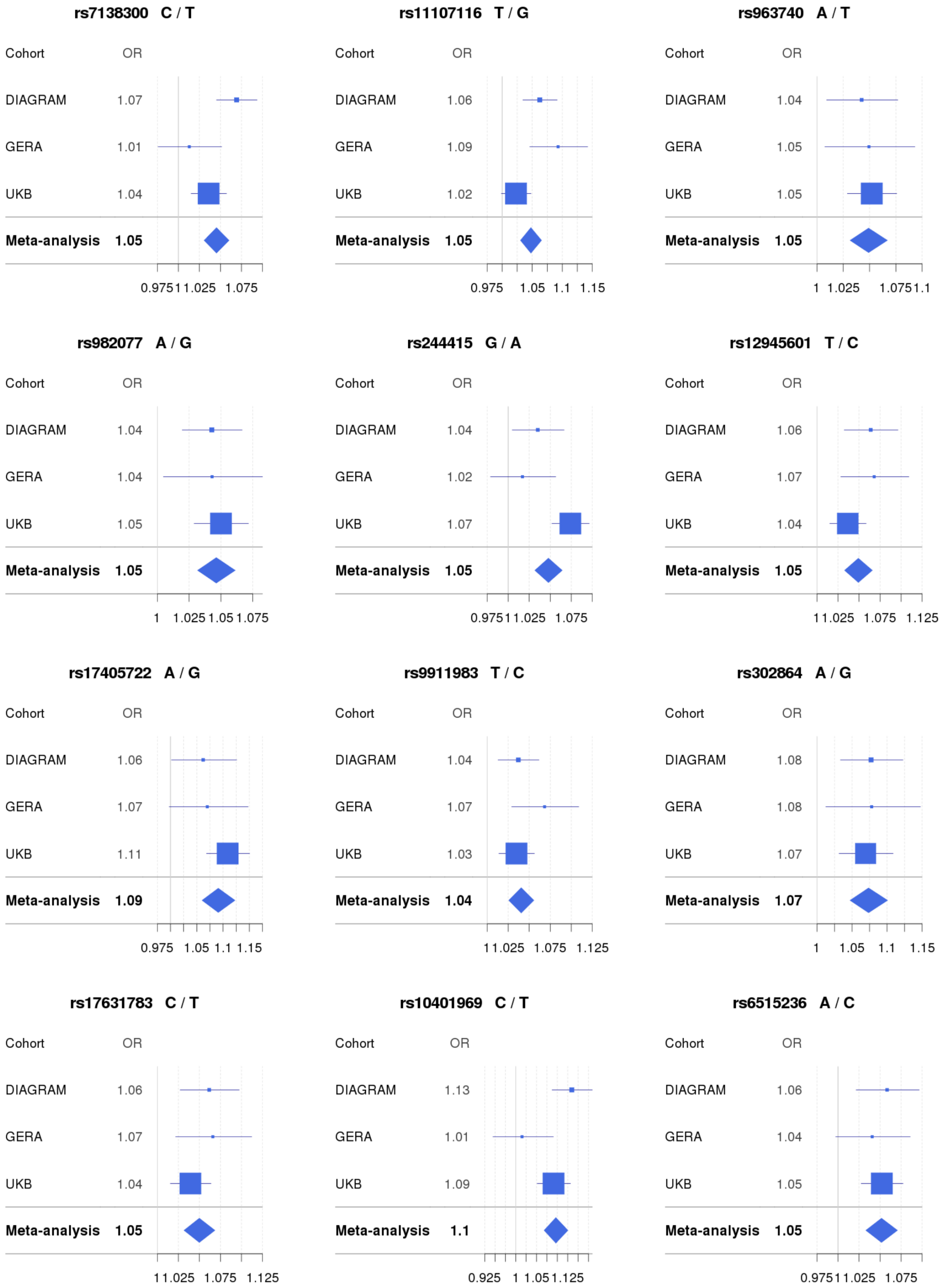
rs7138300 C / T
Cohort OR
DIAGRAM 1.07
GERA 1.01
UKB 1.04
Meta-analysis 1.05
0.975 1 1.025 1.075
rs11107116 T / G
Cohort OR
DIAGRAM 1.06
GERA 1.09
UKB 1.02
Meta-analysis 1.05
0.975 1.05 1.1 1.15
rs963740 A / T
Cohort OR
DIAGRAM 1.04
GERA 1.05
UKB 1.05
Meta-analysis 1.05
1 1.025 1.075 1.1
rs982077 A / G
Cohort OR
DIAGRAM 1.04
GERA 1.04
UKB 1.05
Meta-analysis 1.05
1 1.025 1.05 1.075
rs244415 G / A
Cohort OR
DIAGRAM 1.04
GERA 1.02
UKB 1.07
Meta-analysis 1.05
0.975 1 1.025 1.075
rs12945601 T / C
Cohort OR
DIAGRAM 1.06
GERA 1.07
UKB 1.04
Meta-analysis 1.05
1 1.025 1.075 1.125
rs17405722 A / G
Cohort OR
DIAGRAM 1.06
GERA 1.07
UKB 1.11
Meta-analysis 1.09
0.975 1.05 1.1 1.15
rs9911983 T / C
Cohort OR
DIAGRAM 1.04
GERA 1.07
UKB 1.03
Meta-analysis 1.04
1 1.025 1.075 1.125
rs302864 A / G
Cohort OR
DIAGRAM 1.08
GERA 1.08
UKB 1.07
Meta-analysis 1.07
1 1.05 1.1 1.15
rs17631783 C / T
Cohort OR
DIAGRAM 1.06
GERA 1.07
UKB 1.04
Meta-analysis 1.05
1 1.025 1.075 1.125
rs10401969 C / T
Cohort OR
DIAGRAM 1.13
GERA 1.01
UKB 1.09
Meta-analysis 1.1
0.925 1 1.05 1.125
rs6515236 A / C
Cohort OR
DIAGRAM 1.06
GERA 1.04
UKB 1.05
Meta-analysis 1.05
0.975 1 1.025 1.075

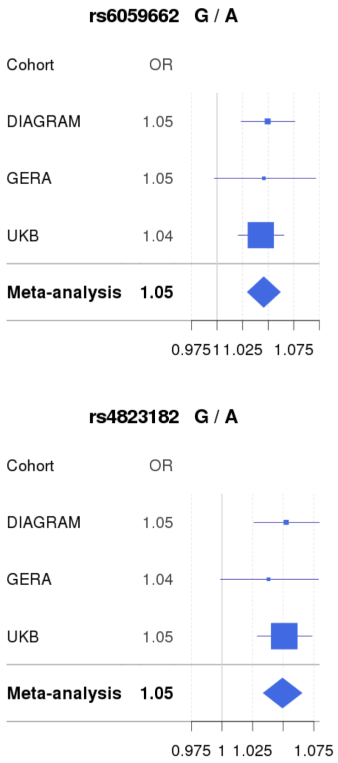

Supplementary Figure 3 Forest plots of the 39 novel loci associated with T2D at $P < 5 \times 10^{-8}$. Error bars represent the 95% confidence intervals. Area of the square (or rhombus) denotes the sample size.

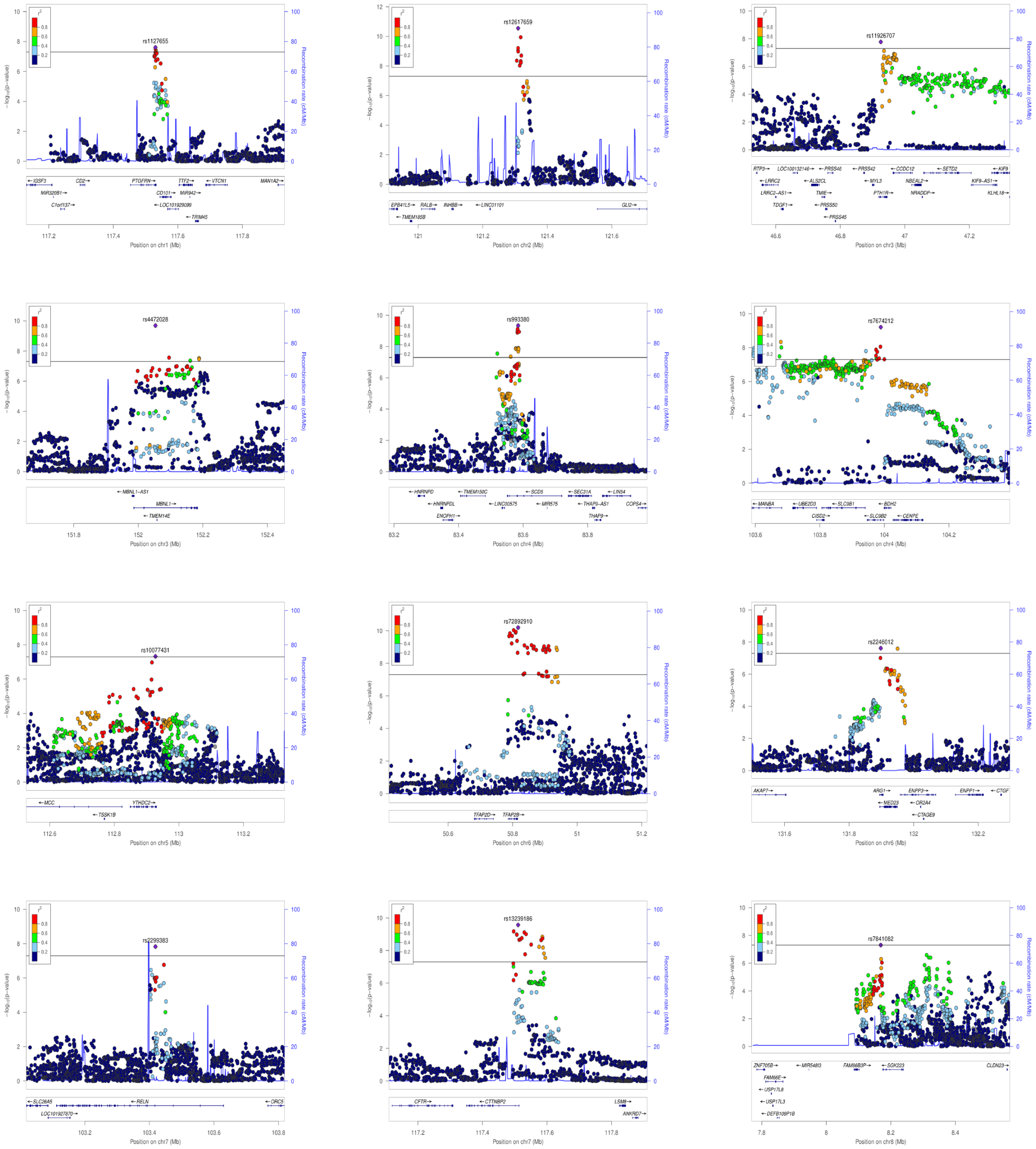
rs1127655
Position on chr1 (Mb)
rs12617659
Position on chr2 (Mb)
rs11926707
Position on chr3 (Mb)
rs4472028
Position on chr3 (Mb)
rs993380
Position on chr4 (Mb)
rs7674212
Position on chr4 (Mb)
rs10077431
Position on chr5 (Mb)
rs72892910
Position on chr6 (Mb)
rs2246012
Position on chr6 (Mb)
rs2299383
Position on chr7 (Mb)
rs13239186
Position on chr7 (Mb)
rs7841082
Position on chr8 (Mb)

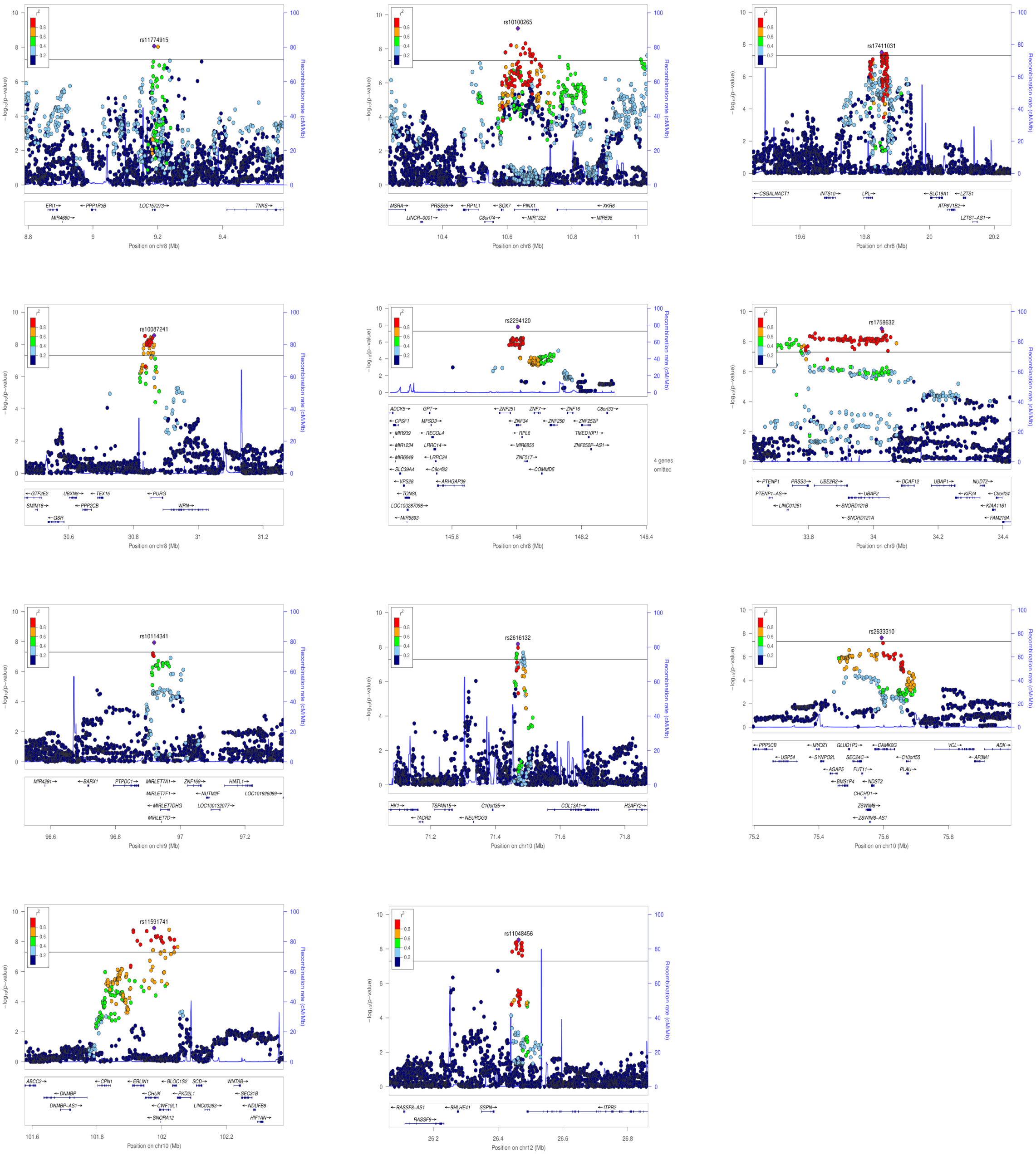
rs11774915
Position on chr8 (Mb)
rs10100265
Position on chr8 (Mb)
rs17411031
Position on chr8 (Mb)
rs10087241
Position on chr8 (Mb)
rs2294120
4 genes omitted
Position on chr8 (Mb)
rs1758632
Position on chr9 (Mb)
rs10114341
Position on chr9 (Mb)
rs2616132
Position on chr10 (Mb)
rs2633310
Position on chr10 (Mb)
rs11591741
Position on chr10 (Mb)
rs11048456
Position on chr12 (Mb)
Recombination rate (cM/Mb)

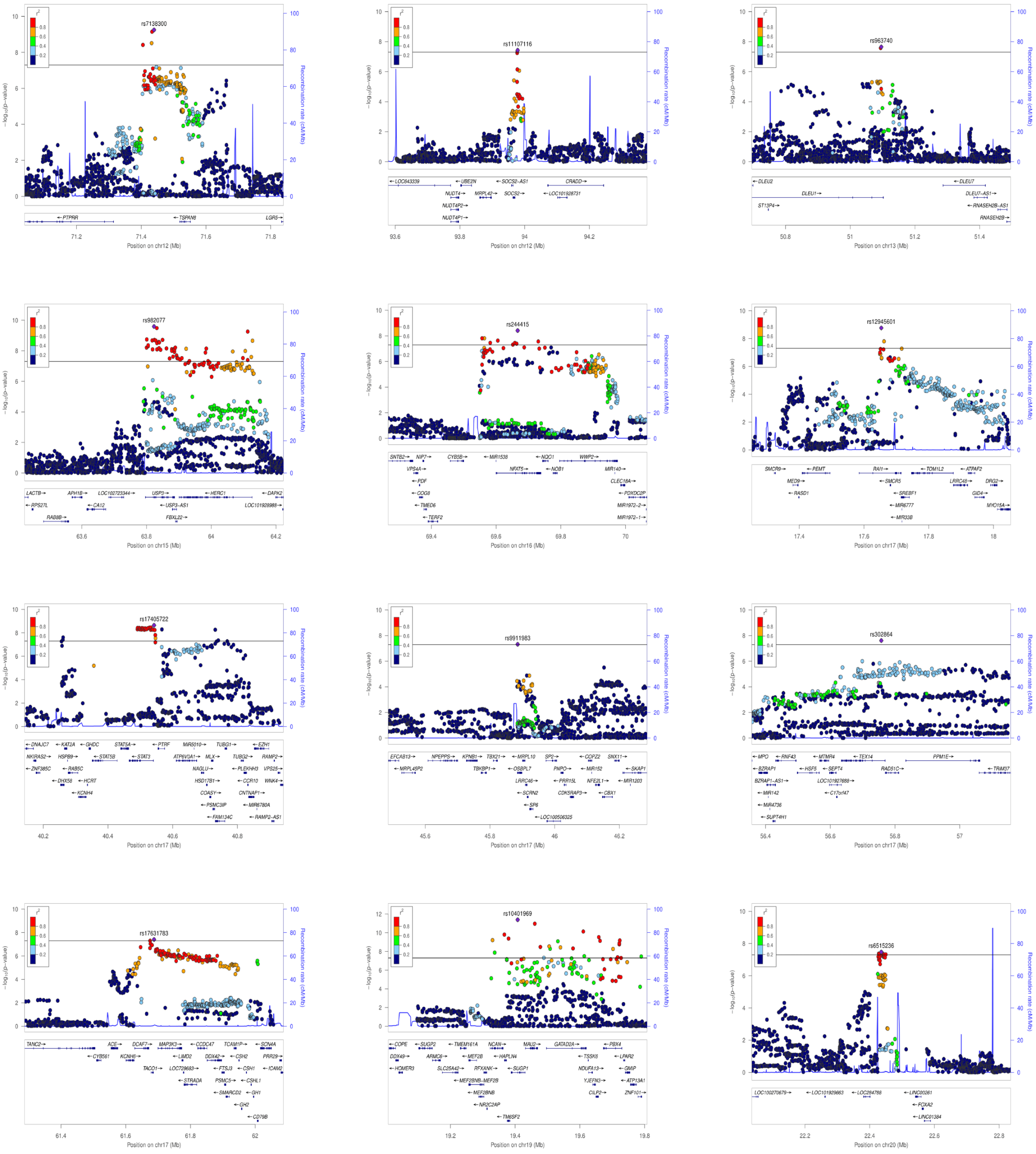
rs7138300
rs11107116
rs963740
rs982077
rs244415
rs12945601
rs17405722
rs9911983
rs302864
rs17631783
rs10401969
rs6515236

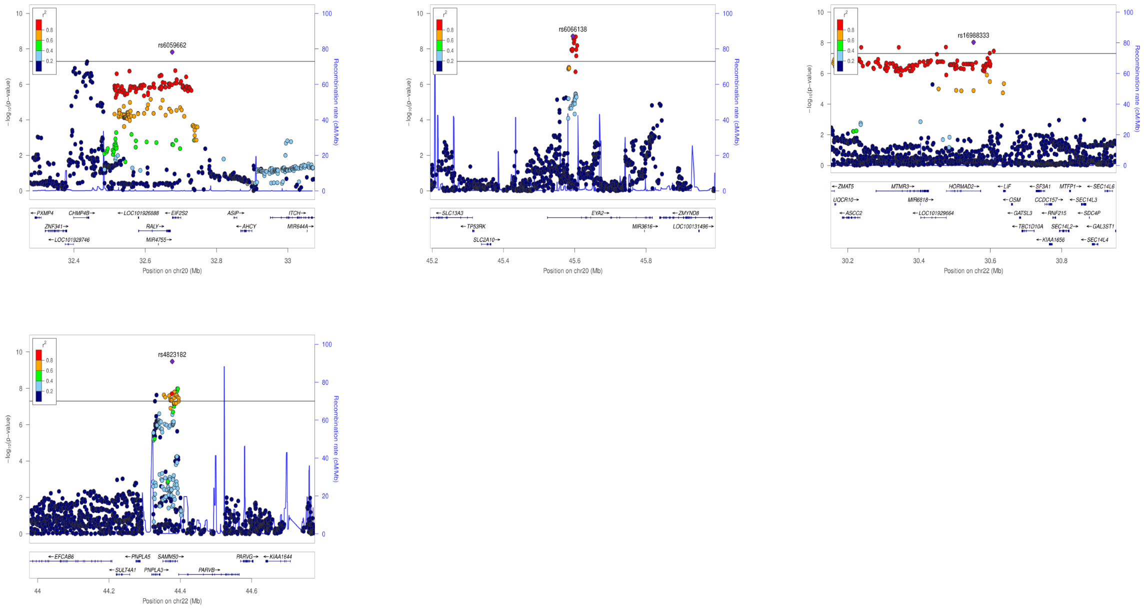

Supplementary Figure 4 Regional plots of the 39 novel loci identified from the meta-analysis at $P < 5 \times 10^{-8}$. Each point represents a SNP passing quality control in the meta-analysis plotted with its P value (on a $-\log_{10}$ scale) against its genomic position (NCBI Build 37). In each plot, the lead SNP is represented by the purple symbol. The color coding of all other SNPs indicate LD with the lead SNP (estimated by CEU r^2 values from Phase I 1000 Genomes): red, $r^2 \geqslant 0.8$; gold, $0.6 \leqslant r^2 < 0.8$; green, $0.4 \leqslant r^2 < 0.6$; cyan, $0.2 \leqslant r^2 < 0.4$; blue, $r^2 < 0.2$; grey, r^2 unknown. Recombination rates are estimated from Phase I 1000 Genomes, and gene annotations are taken from the UCSC genome browser.

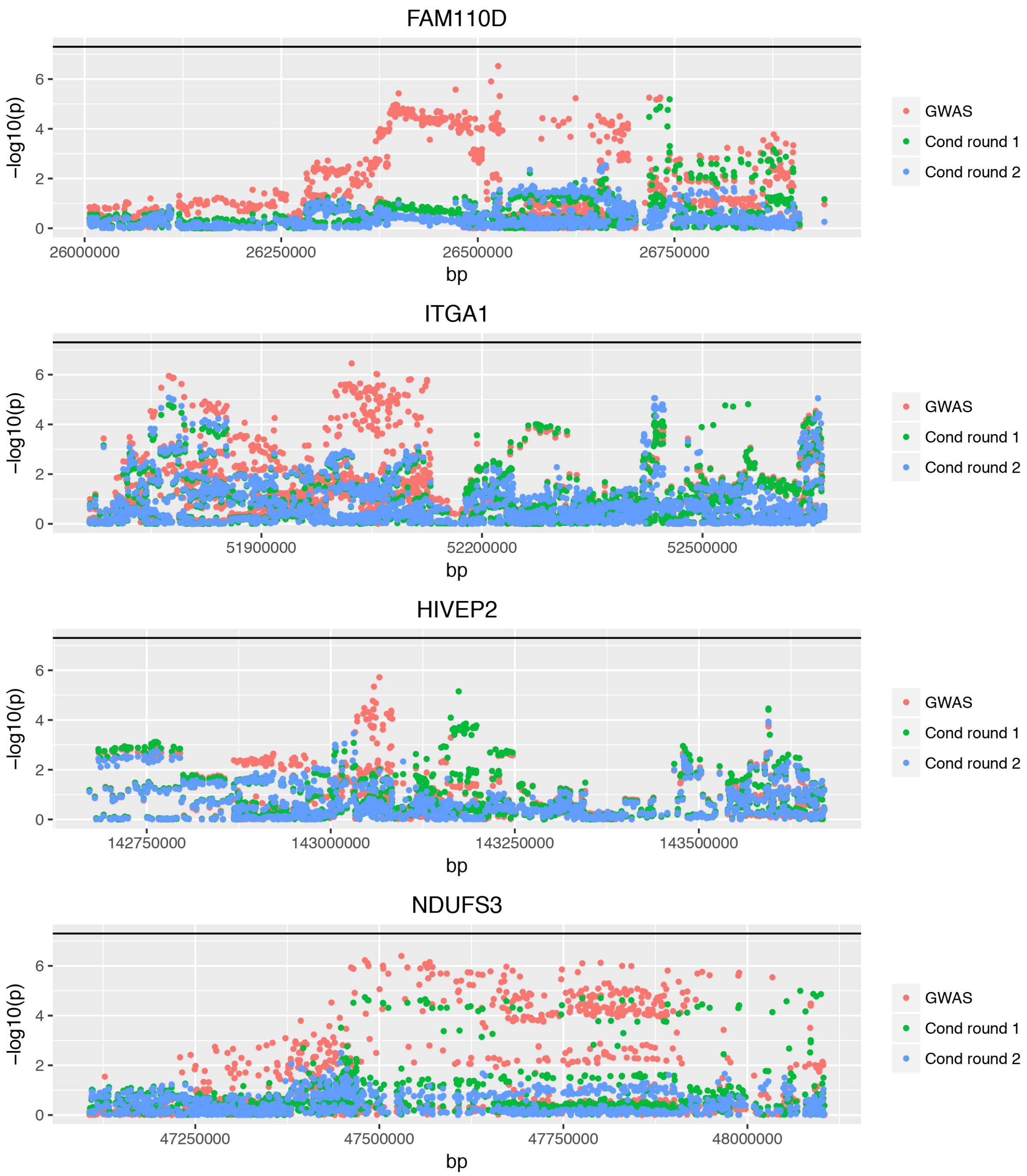

Supplementary Figure 5 Conditional association analysis at the four novel loci identified by the GCTA-fastBAT analysis.

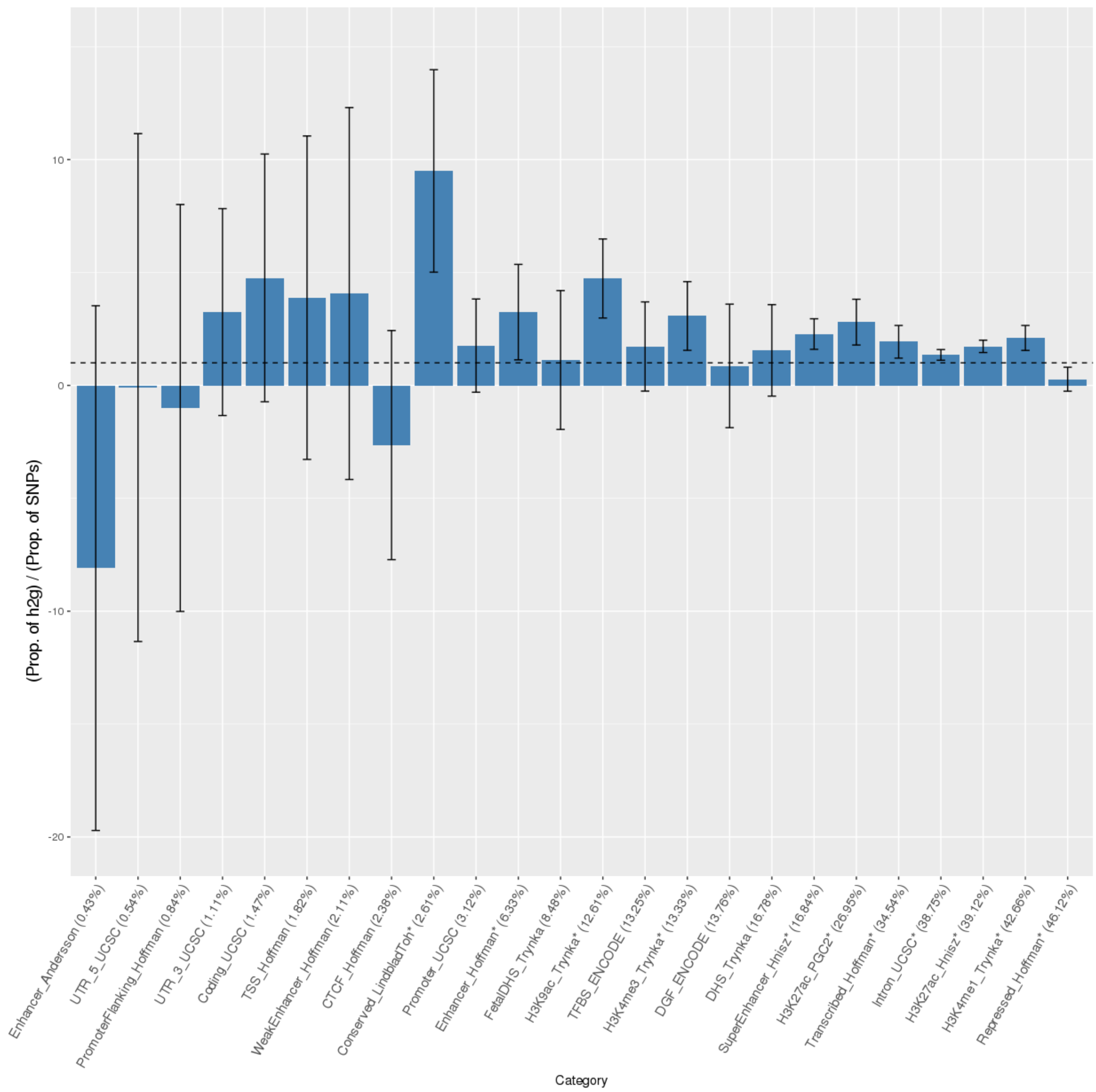

Supplementary Figure 6 Enrichment of the variance explained by SNPs for T2D in 24 functional annotations. Shown are the results from the LD score regression based functional partitioning analysis[21]. The 24 functional annotations are defined in Finucane *et al.*[21]. Annotations are ordered by proportion of SNPs. Error bar represents the 95% confidential interval around the estimate of enrichment, and the asterisk indicates a significant estimate at $P < 0.05$ after Bonferroni correction for 24 tests. CTCF, CCCTC-binding factor. DGF, digital genomic footprint. DHS, DNase I hypersensitive site. TFBS, transcription factor binding site. TSS, transcription start site.

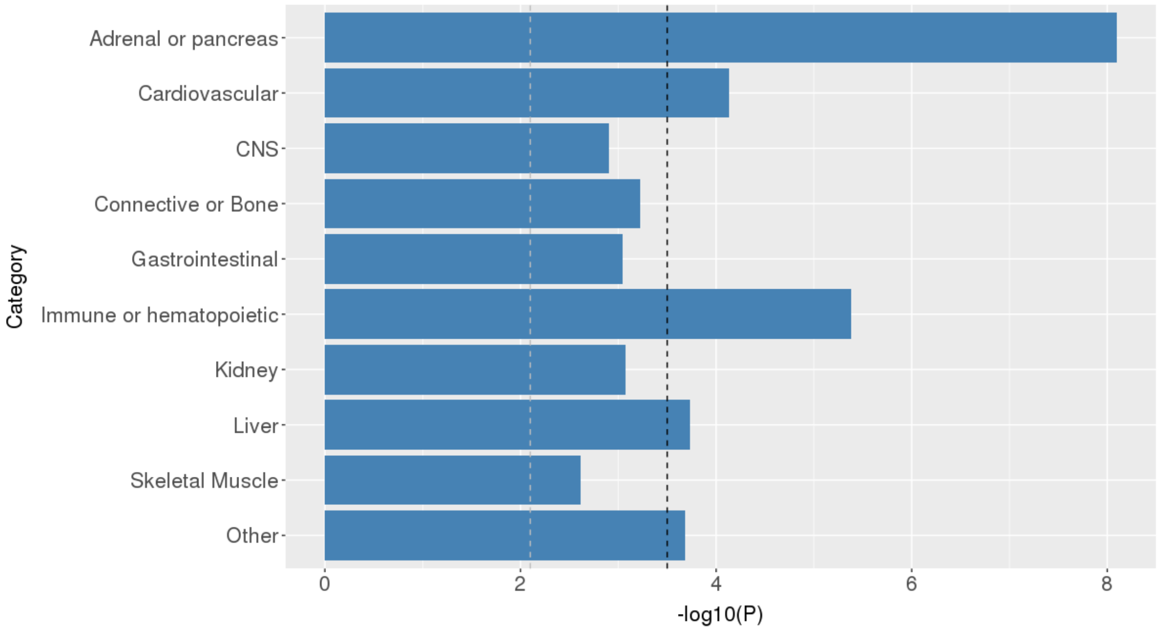

Supplementary Figure 7 Enrichment of the variance explained by SNPs for T2D in 10 cell type groups. Shown are the results from the LD score regression based functional partitioning analysis[21]. The black dashed lines at $-\log_{10}(P) = 3.6$ is the significance level after Bonferroni correction. The grey dashed lines at $-\log_{10}(P) = 2.3$ is the threshold at FDR < 0.05.

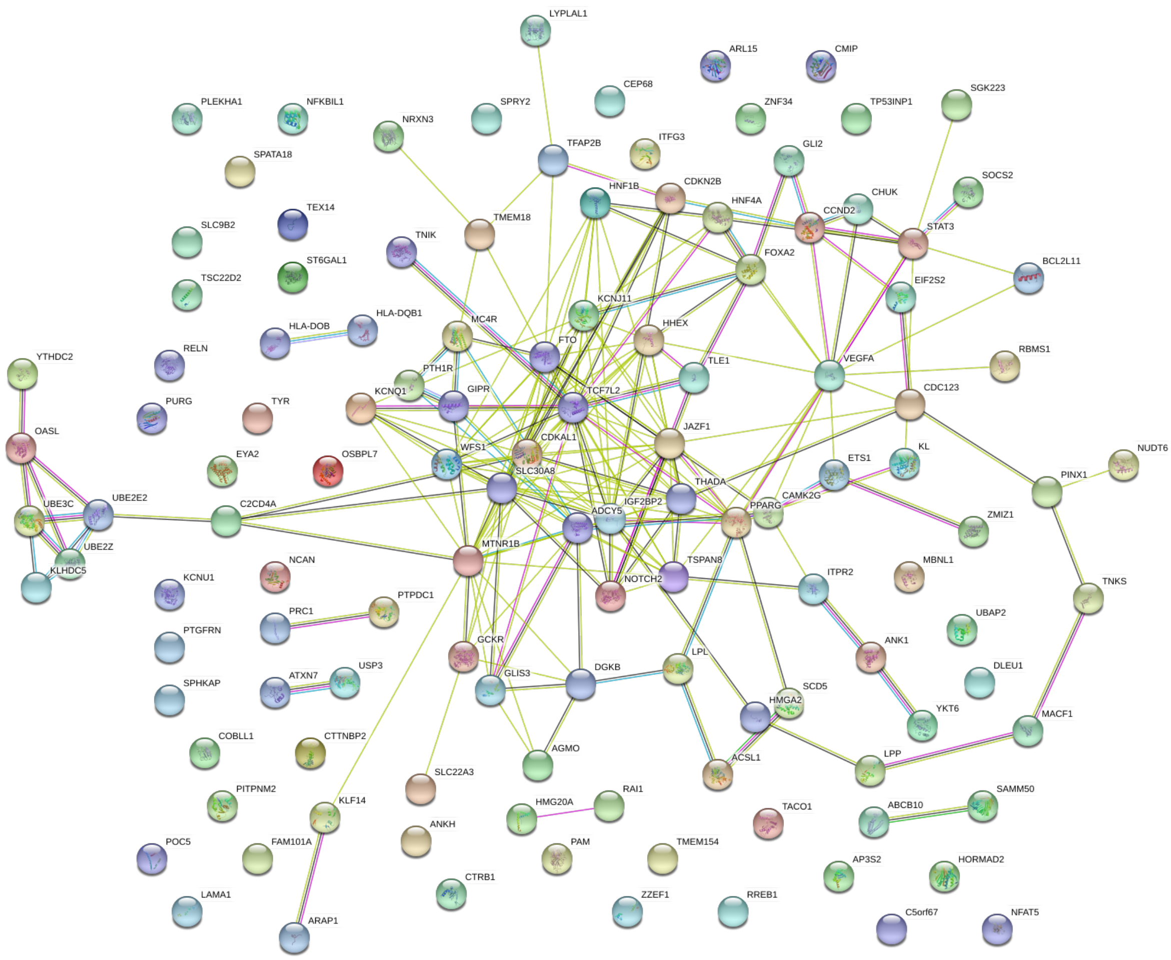

Supplementary Figure 8 Enrichment of the T2D-associted genes in protein-protein interaction network. There are 123 nodes and 210 edges in total generated by STRING v10. Network nodes represent proteins. Small node represents protein of unknown 3D structure while large node represents that the 3D structure is known or predicted. Edges represent protein-protein associations. Dark purple edge represents experimentally determined interaction, light purple edge represents protein homology, and yellow edge represents interaction from text-mining.

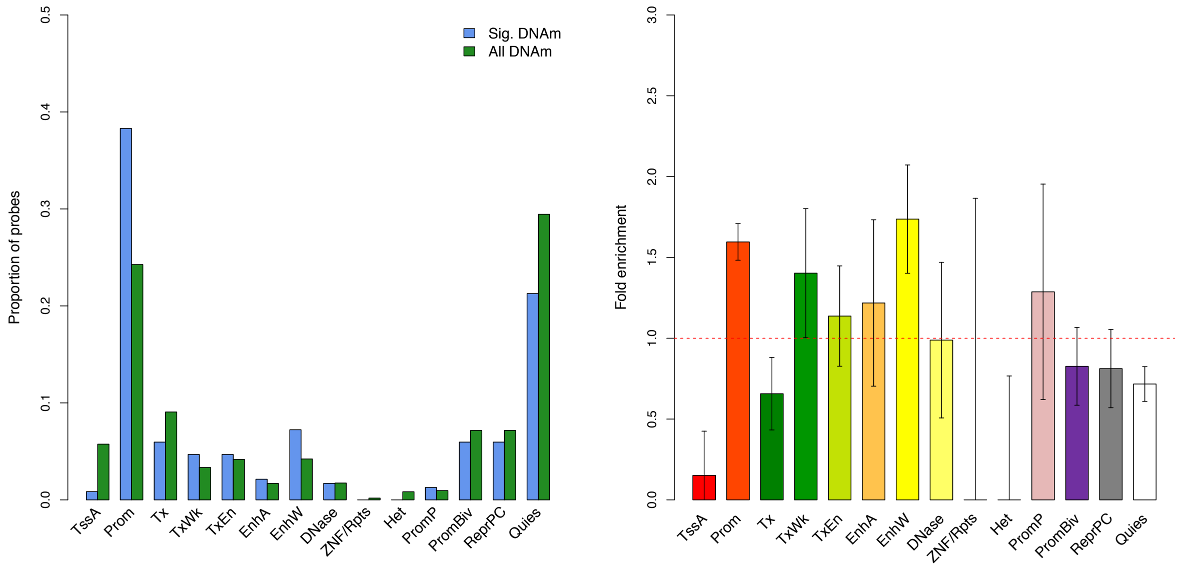

Supplementary Figure 9 Enrichment of the 235 T2D-associated DNAm sites in functional categories. a) Distribution of the T2D-associated DNAm probes ("Sig. DNAm", blue) across the 14 functional categories in comparison with that of all DNAm probes in the data ("All DNAm", green). b) Fold enrichment: a comparison of the T2D-associated probes with the same number of probes sampled repeatedly at random with the variance of each probe matched. Error bar represents the standard error of an estimate obtained from 500 random samples. The 14 functional annotation categories are: TssA, active transcription start site; Prom, upstream/downstream TSS promoter; Tx, actively transcribed state; TxWk, weak transcription; TxEn, transcribed and regulatory Prom/Enh; EnhA, active enhancer; EnhW, weak enhancer; DNase, primary DNase; ZNF/Rpts, state associated with zinc finger protein genes; Het, constitutive heterochromatin; PromP, Poised promoter; PromBiv, bivalent regulatory states; ReprPC, repressed Polycomb states; and Quies, a quiescent state.

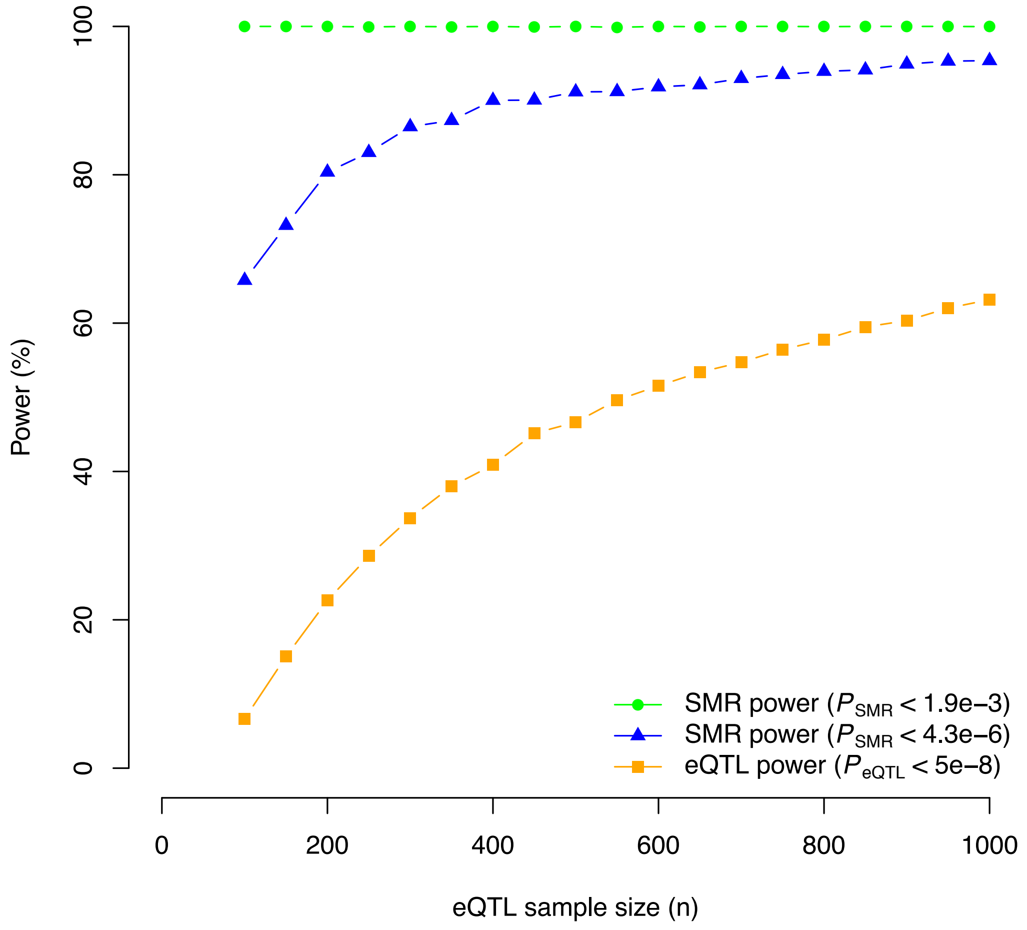

Supplementary Figure 10 The power of the SMR test with varied sample size of eQTL study. The simulation method is described in the **Supplementary Note 9**. The sample size of the simulated disease trait was 659,316 (with a prevalence of 10%). Here, we investigated the SMR replication power at $P < 1.9 \times 10^{-3}$ (0.05/27, the number genes identified by SMR and HEIDI using the eQTLGen data) and the SMR discovery power at $P < 4.3 \times 10^{-6}$ (0.05/11,743, the number of probes in the eQTLGen data). The simulation result showed that SMR power was high even with small sample size eQTL data (e.g. GTEx data), but its discovery power was restricted by the power of eQTL detection. When $n_{\text{eQTL}} = 400$, i.e., the upper limit of n_{eQTL} in the GTEx-AALP data, the discovery power at $P_{\text{SMR}} < 4.3 \times 10^{-6}$ was closet to 90% and the gene discoveries would only depend on the eQTL power (43%). Note that the replication power ($P_{\text{SMR}} < 1.9 \times 10^{-3}$) was much larger than the discovery power ($P_{\text{SMR}} < 4.3 \times 10^{-6}$) even if n_{eQTL} was relatively small.

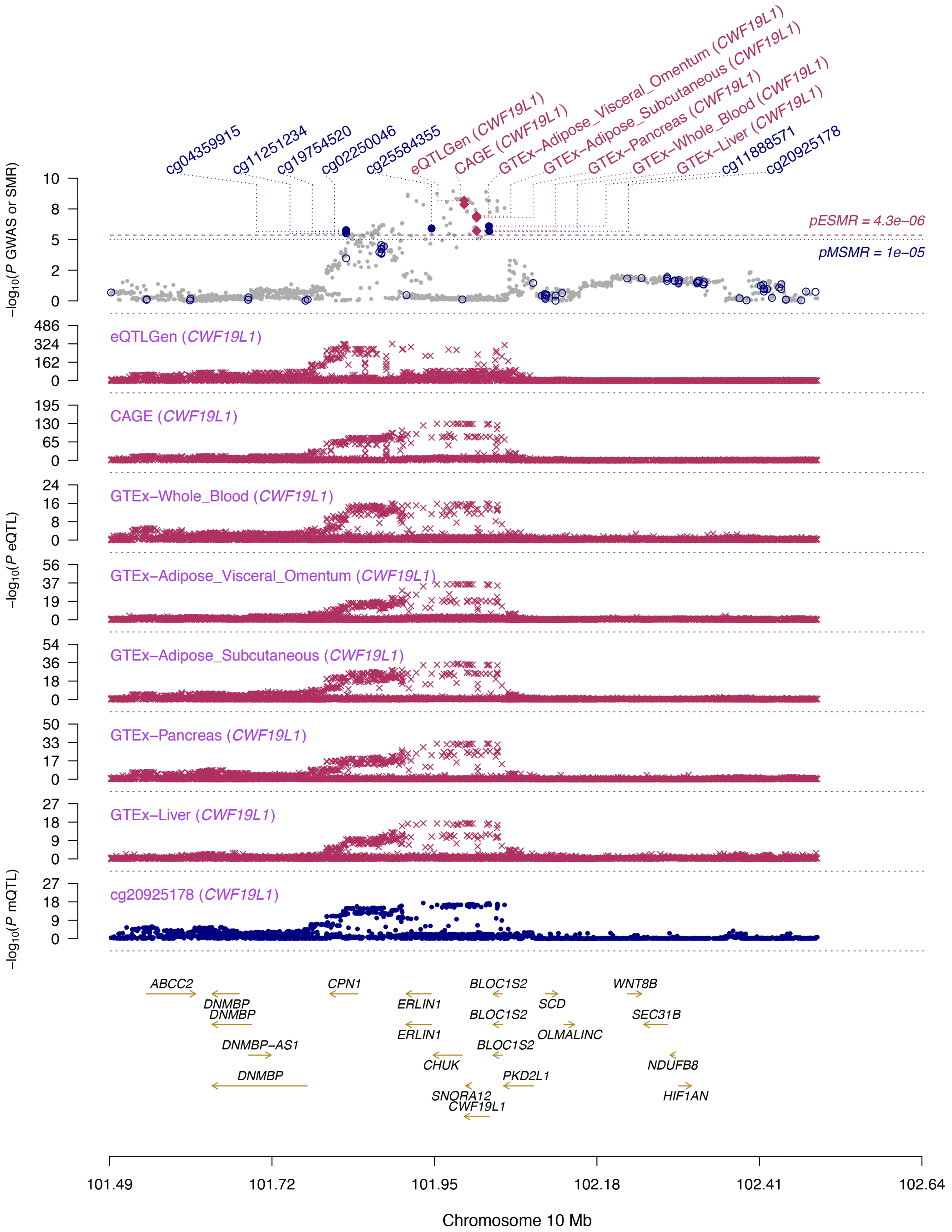

Supplementary Figure 11 Association signals of the expression level of *CWF19L1* with the cis-SNPs in eQTLGen and CAGE in comparison with those in five GTEx tissues. Shown are the results from the SMR analysis that integrates data from GWAS, eQTL and mQTL studies. The top plot shows -$\log_{10}$(P-value) from our GWAS meta-analysis. Red diamonds and blue circles represent -$\log_{10}$(P-value) from the SMR tests for associations of gene expression and DNA methylation probes with T2D, respectively. Solid diamonds and circles represent the probes not rejected by the HEIDI test. The second plot shows -$\log_{10}$(P-value) of SNP associations with gene expression probes (tagging *CWF19L1*) in eQTLGen, CAGE and five GTEx tissues. The third plot shows -$\log_{10}$(P-value) of SNP associations with a DNA methylation probe cg20925178.

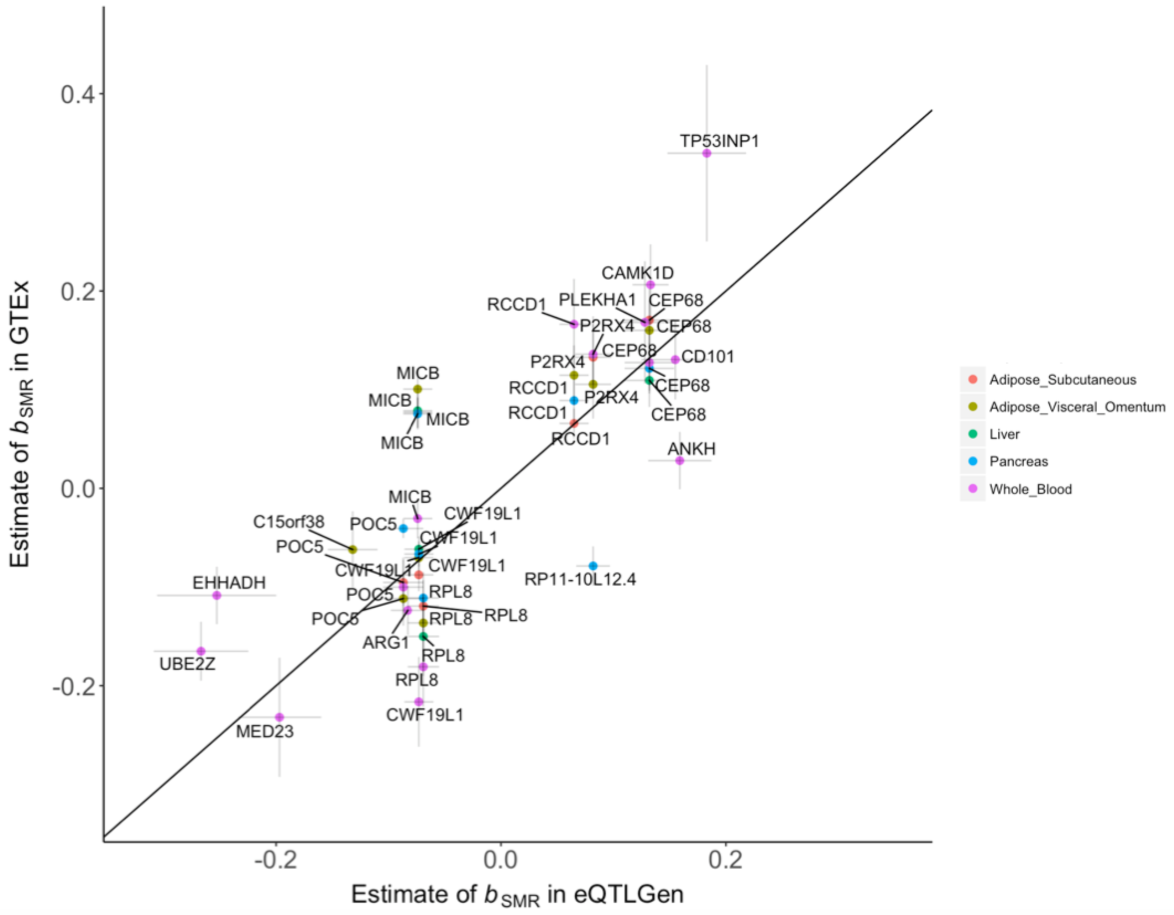

Supplementary Figure 12 Estimates of SMR effects in eQTLGen vs. those in the five GTEx tissues. The SMR effect (b_{SMR}) is defined as the effect of the expression level of a gene on T2D risk[28]. Shown are the 18 genes detected using the eQTLGen data with b_{SMR} estimated in eQTLGen plotted against those estimated in the GTEx tissues (including pancreas, liver and/or adiposes). The MHC region was not included in the analysis. Each dot represents a gene with colors indicating different tissues in GTEx. Error bar represents the standard error for an estimate of b_{SMR}. The correlation of b_{SMR} estimates between eQTLGen and GTEx is 0.80.

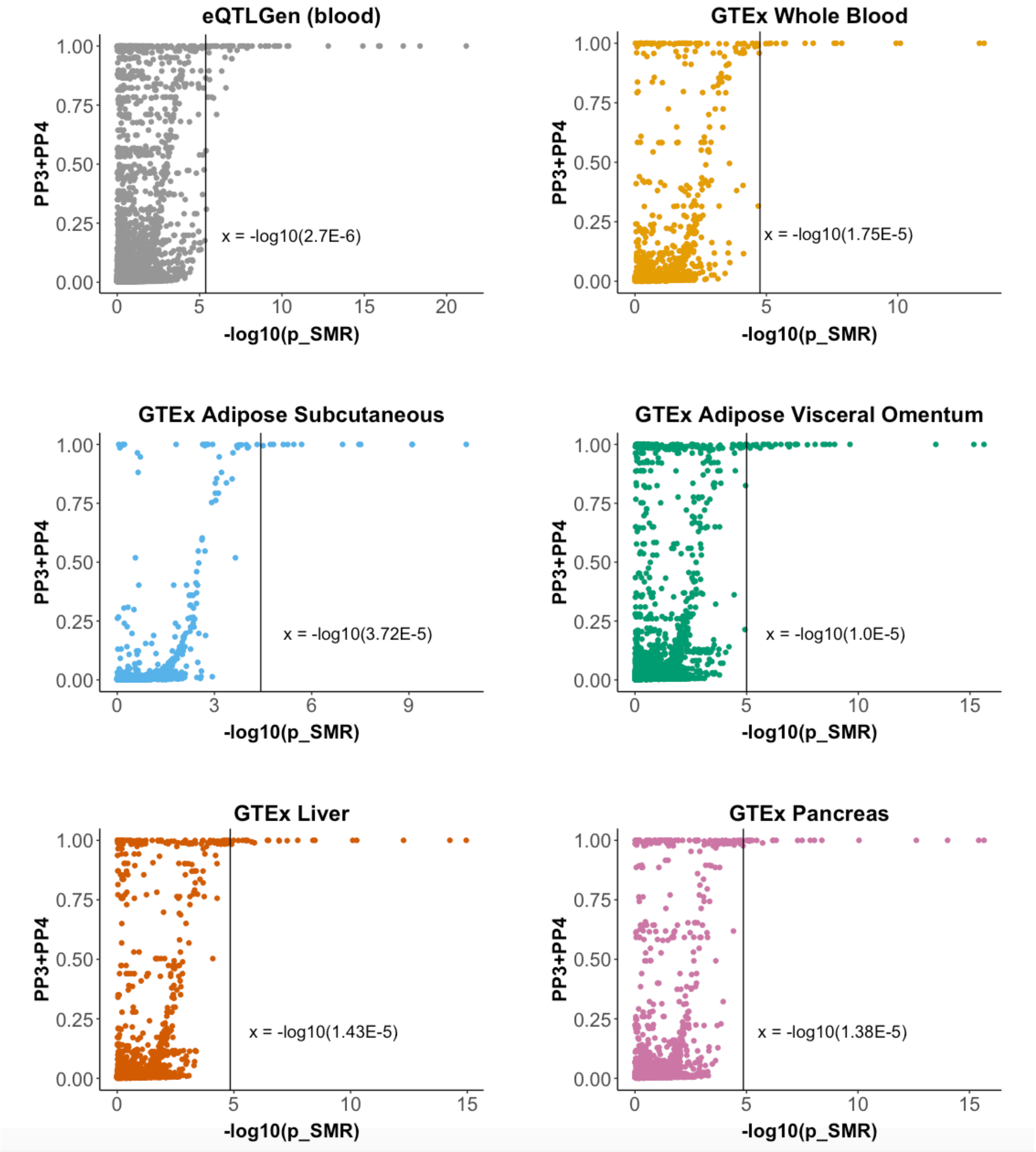

Supplementary Figure 13 Comparison of the p-values from the SMR analysis with the posterior probabilities (PP) from the COLOC analysis. PP3 represents the posterior probability of the hypothesis that there are two SNPs (likely in LD) associated with gene expression and T2D, respectively. PP4 represents the posterior probability of the hypothesis that there is one shared SNP associated with gene expression and T2D. The result showed that most genes that passed the genome-wide significant threshold in the SMR test also had extremely high PP of associations with T2D from the COLOC analysis, suggesting a good concordance between the two methods.

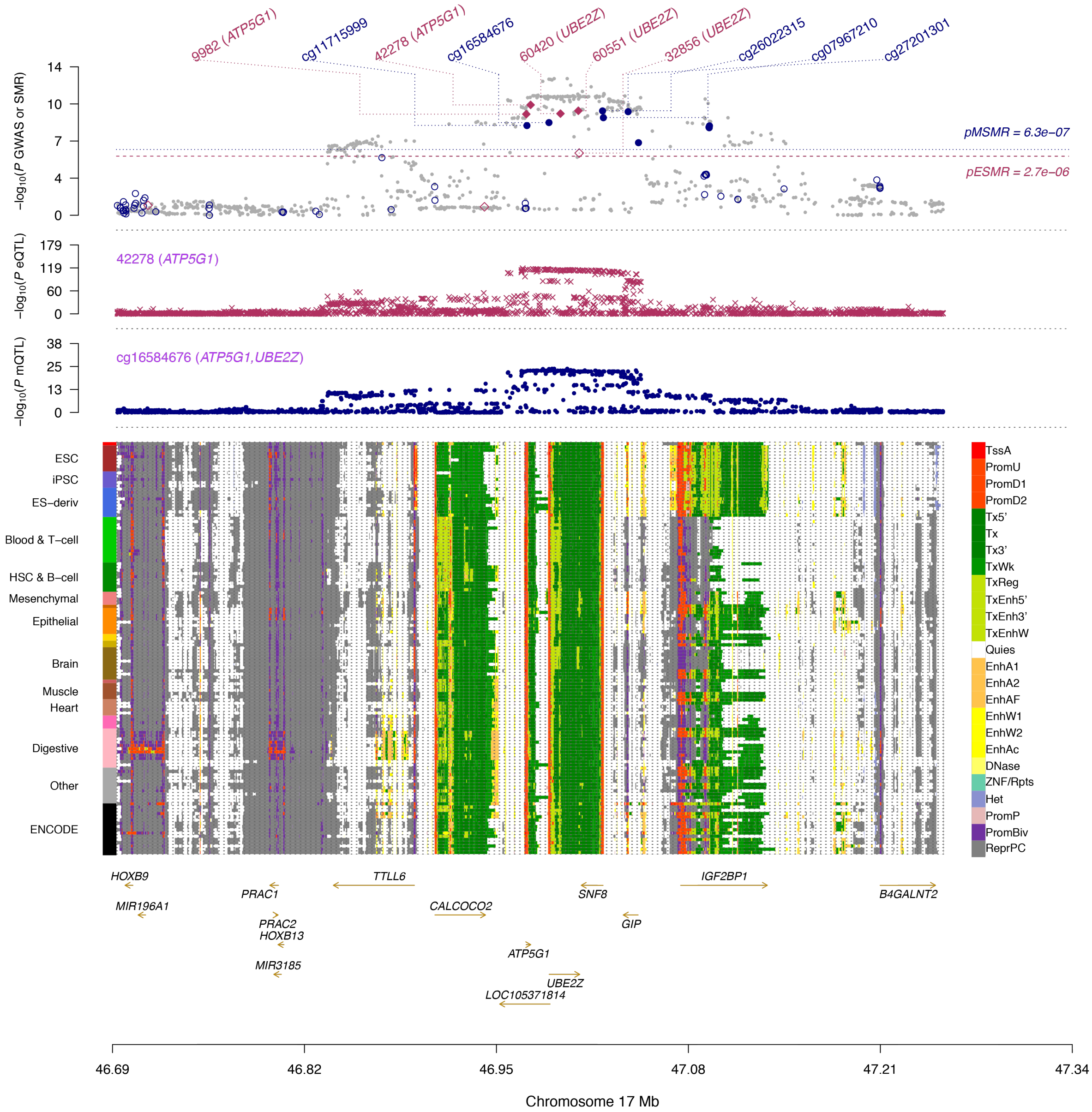

Supplementary Figure 14a Prioritizing genes and regulatory elements at the *ATP5G1* locus. Shown are the results from the SMR analysis that integrates data from GWAS, eQTL and mQTL studies. The top plot shows -$\log_{10}$(P-value) of SNPs from the GWAS meta-analysis for T2D. Red diamonds and blue circles represent -$\log_{10}$(P-value) from the SMR tests for associations of gene expression and DNAm probes with T2D, respectively. Solid diamonds and circles represent the probes not rejected by the HEIDI test. The second plot shows -$\log_{10}$(P-value) of the SNP association for gene expression probe 42278 (tagging *ATP5G1*). The third plot shows -log10(P-value) of the SNP association with DNAm probe cg16584676. The bottom plot shows 25 chromatin state annotations (indicated by colours) of 127 samples from Roadmap Epigenomics Mapping Consortium (REMC) for different primary cells and tissue types (rows).

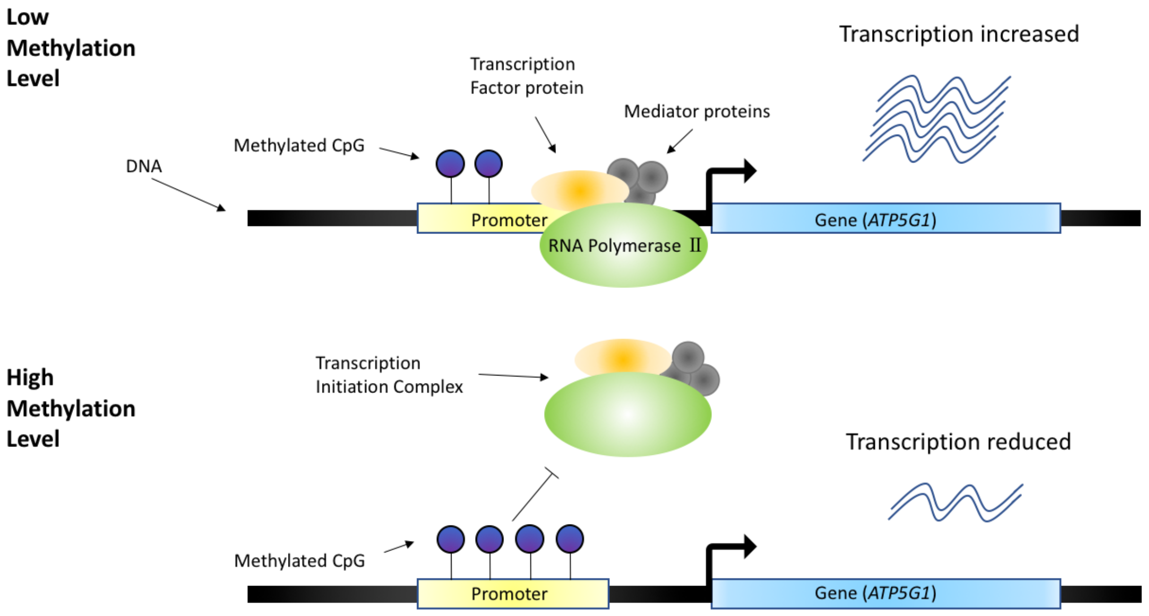

Supplementary Figure 14b Hypothesized mechanism of how DNA methylation affect the expression level of *ATP5G1*. When the methylation level of the promoter region is low, the RNA polymerase II binds to the promoter region with the assistance from transcription factors (TF), and initiate the transcription. However, if the promoter region is highly methylated, it would obstruct the binding of RNA polymerase II to promoter region, which leads to the reduction of transcription.

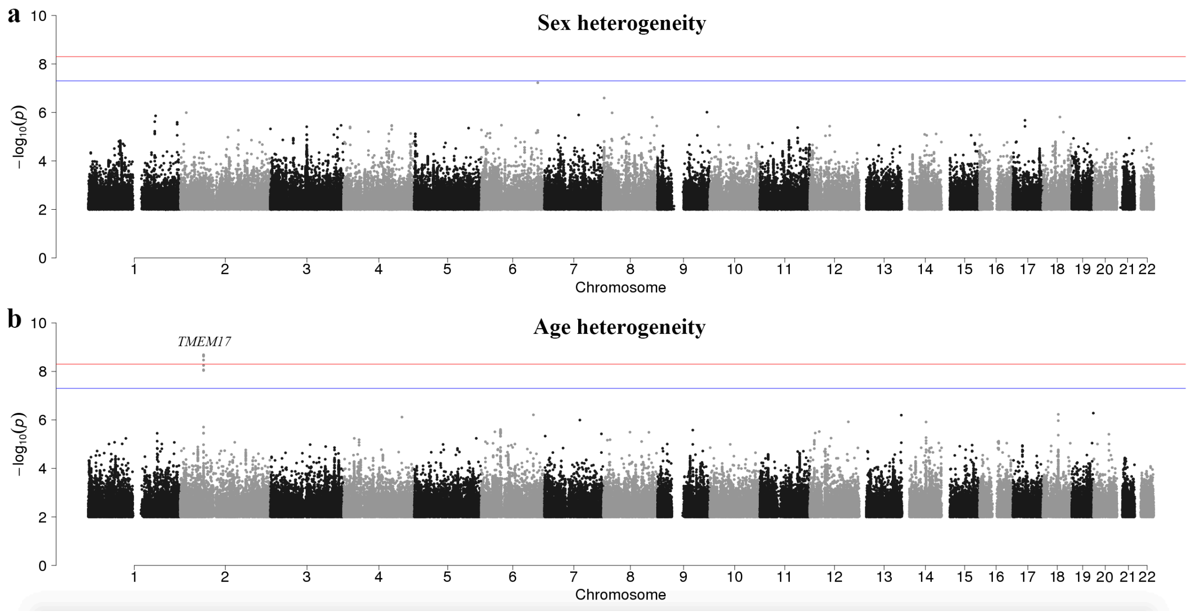

Supplementary Figure 15 Heterogeneity in SNP effect between sex (or age) groups in UKB. Shown are the Manhattan plots from the heterogeneity tests between sex (or age) groups for all 18,138,214 variants (including the rare variants) (**Supplementary Note 11**). The x-axis is the chromosome number and the y-axis is the $-\log_{10}$ of heterogeneity *P*-value. The blue lines represent a genome-wide significance level at $P < 5\times10^{-8}$, and the red lines represent a threshold of 5×10^{-9} (as suggested by Wu *et al.*[41] for GWAS with both common and rare variants from imputation). SNPs with $P_{\text{heter}} > 0.01$ are omitted.

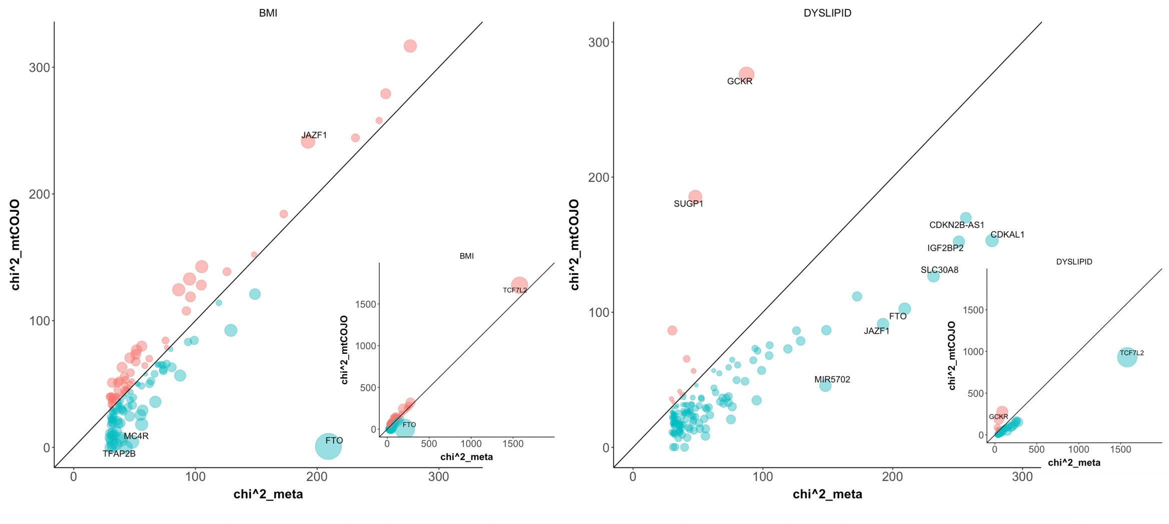

Supplementary Figure 16 Test-statistics at the 139 T2D loci conditioning on BMI or Dyslipidaemia by a GCTA-mtCOJO analysis vs. those from the original meta-analysis. Each circle represents a locus. Shown on the x-axis are the chi-squared statistics from the original meta-analysis and those on the y-axis are the chi-square statistics from the mtCOJO analysis. The size of a circle reflects the difference in chi-square statistic between meta-analysis and mtCOJO analysis. The loci with relatively large differences are labelled with the names of their nearest genes.

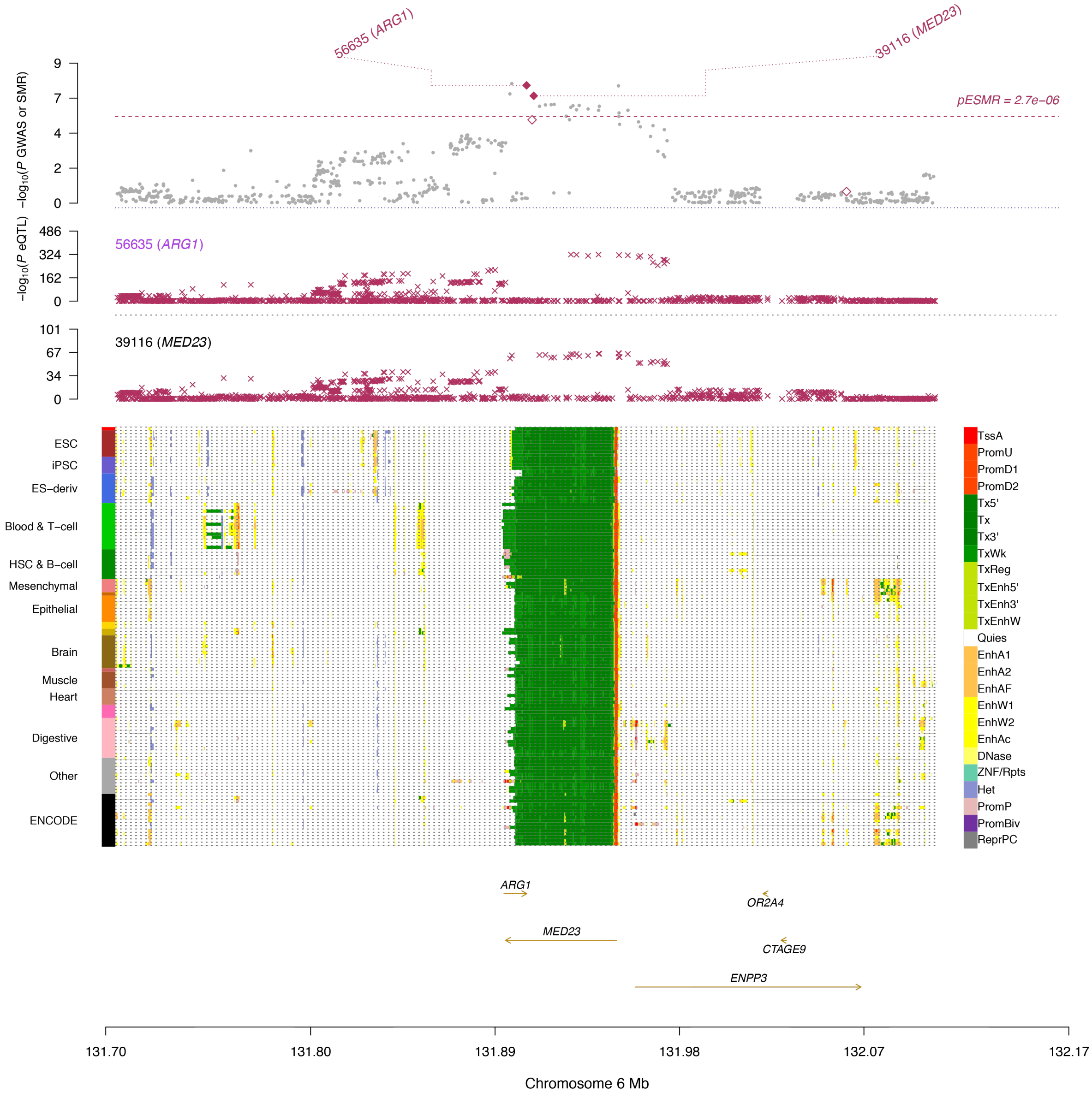

Supplementary Figure 17 Prioritizing genes and regulatory elements at the *ARG1* locus. Shown are the results from the SMR analysis that integrates data from GWA and eQTL studies. The top plot shows -$\log_{10}$(P-value) of SNPs from the GWAS meta-analysis for T2D. Red diamonds represent -$\log_{10}$(P-value) from the SMR tests for associations of gene expression probes with T2D. Solid diamonds represent the probes not rejected by the HEIDI test. The second plot shows -$\log_{10}$(P-value) of the SNP association for gene expression probe 56635 (tagging *ARG1*) and 39166 (tagging *MED23*). The bottom plot shows 25 chromatin state annotations (indicated by colours) of 127 samples from Roadmap Epigenomics Mapping Consortium (REMC) for different primary cells and tissue types (rows).

References

1. Pasaniuc, B. *et al.* Fast and accurate imputation of summary statistics enhances evidence of functional enrichment. *Bioinformatics* **30**, 2906-2914 (2014).
2. Bulik-Sullivan, B.K. *et al.* LD Score regression distinguishes confounding from polygenicity in genome-wide association studies. *Nature Genetics* **47**, 291-295 (2015).
3. Bulik-Sullivan, B. *et al.* An atlas of genetic correlations across human diseases and traits. *Nat Genet* **47**, 1236-41 (2015).
4. Chen, G.B. *et al.* Across-cohort QC analyses of GWAS summary statistics from complex traits. *Eur J Hum Genet* **25**, 137-146 (2016).
5. Loh, P.R. *et al.* Efficient Bayesian mixed-model analysis increases association power in large cohorts. *Nat Genet* **47**, 284-90 (2015).
6. Loh, P.R., Kichaev, G., Gazal, S., Schoech, A.P. & Price, A.L. Mixed-model association for biobank-scale datasets. *Nat Genet* (2018).
7. Zhou, W. *et al.* Efficiently controlling for case-control imbalance and sample relatedness in large-scale genetic association studies. *bioRxiv*, 212357 (2017).
8. Malakar, P. *et al.* Insulin receptor alternative splicing is regulated by insulin signaling and modulates beta cell survival. *Sci Rep* **6**, 31222 (2016).
9. Sen, S. *et al.* Muscleblind-like 1 (Mbnl1) promotes insulin receptor exon 11 inclusion via binding to a downstream evolutionarily conserved intronic enhancer. *J Biol Chem* **285**, 25426-37 (2010).
10. Velayos, T. *et al.* An Activating Mutation in STAT3 Results in Neonatal Diabetes Through Reduced Insulin Synthesis. *Diabetes* **66**, 1022-1029 (2017).
11. Saarimaki-Vire, J. *et al.* An Activating STAT3 Mutation Causes Neonatal Diabetes through Premature Induction of Pancreatic Differentiation. *Cell Rep* **19**, 281-294 (2017).
12. Perez-Diaz, S. *et al.* Polymerase I and transcript release factor (PTRF) regulates adipocyte differentiation and determines adipose tissue expandability. *FASEB J* **28**, 3769-79 (2014).
13. Aboulaich, N., Ortegren, U., Vener, A.V. & Stralfors, P. Association and insulin regulated translocation of hormone-sensitive lipase with PTRF. *Biochem Biophys Res Commun* **350**, 657-61 (2006).
14. Ozcan, L. *et al.* Activation of calcium/calmodulin-dependent protein kinase II in obesity mediates suppression of hepatic insulin signaling. *Cell Metab* **18**, 803-15 (2013).
15. Wolfrum, C., Asilmaz, E., Luca, E., Friedman, J.M. & Stoffel, M. Foxa2 regulates lipid metabolism and ketogenesis in the liver during fasting and in diabetes. *Nature* **432**, 1027-32 (2004).
16. Bakshi, A. *et al.* Fast set-based association analysis using summary data from GWAS identifies novel gene loci for human complex traits. *Sci Rep* **6**, 32894 (2016).
17. Yang, J. *et al.* Conditional and joint multiple-SNP analysis of GWAS summary statistics identifies additional variants influencing complex traits. *Nature Genetics* **44**, 369-U170 (2012).
18. International Schizophrenia Consortium *et al.* Common polygenic variation contributes to risk of schizophrenia and bipolar disorder. *Nature* **460**, 748-52 (2009).
19. Wray, N.R., Yang, J., Goddard, M.E. & Visscher, P.M. The genetic interpretation of area under the ROC curve in genomic profiling. *PLoS Genet* **6**, e1000864 (2010).
20. Robinson, M.R. *et al.* Genetic evidence of assortative mating in humans. *Nature Human Behaviour* **1**, 0016 (2017).
21. Finucane, H.K. *et al.* Partitioning heritability by functional annotation using genome-wide association summary statistics. *Nat Genet* **47**, 1228-35 (2015).
22. Lindblad-Toh, K. *et al.* A high-resolution map of human evolutionary constraint using 29 mammals. *Nature* **478**, 476-82 (2011).
23. Trynka, G. *et al.* Chromatin marks identify critical cell types for fine mapping complex trait variants. *Nat Genet* **45**, 124-30 (2013).
24. de Leeuw, C.A., Mooij, J.M., Heskes, T. & Posthuma, D. MAGMA: generalized gene-set analysis of GWAS data. *PLoS Comput Biol* **11**, e1004219 (2015).

25. Szklarczyk, D. *et al.* STRING v10: protein-protein interaction networks, integrated over the tree of life. *Nucleic Acids Res* **43**, D447-52 (2015).
26. Roadmap Epigenomics Consortium *et al.* Integrative analysis of 111 reference human epigenomes. *Nature* **518**, 317-30 (2015).
27. Wu, Y. *et al.* Integrative analysis of omics summary data reveals putative mechanisms underlying complex traits. *Nat Commun* **9**, 918 (2018).
28. Zhu, Z.H. *et al.* Integration of summary data from GWAS and eQTL studies predicts complex trait gene targets. *Nature Genetics* **48**, 481-7 (2016).
29. Walford, G.A. *et al.* Genome-Wide Association Study of the Modified Stumvoll Insulin Sensitivity Index Identifies BCL2 and FAM19A2 as Novel Insulin Sensitivity Loci. *Diabetes* **65**, 3200-11 (2016).
30. Prokopenko, I. *et al.* A central role for GRB10 in regulation of islet function in man. *PLoS Genet* **10**, e1004235 (2014).
31. Global Lipids Genetics Consortium *et al.* Discovery and refinement of loci associated with lipid levels. *Nat Genet* **45**, 1274-83 (2013).
32. Liang, Q. *et al.* Application of citrate as a tricarboxylic acid (TCA) cycle intermediate, prevents diabetic-induced heart damages in mice. *Iran J Basic Med Sci* **19**, 43-8 (2016).
33. Schrauwen, P. & Hesselink, M.K. Reduced tricarboxylic acid cycle flux in type 2 diabetes mellitus? *Diabetologia* **51**, 1694-7 (2008).
34. Morris, A.P. *et al.* Large-scale association analysis provides insights into the genetic architecture and pathophysiology of type 2 diabetes. *Nat Genet* **44**, 981-90 (2012).
35. DIAbetes Genetics Replication And Meta-analysis Consortium *et al.* Genome-wide trans-ancestry meta-analysis provides insight into the genetic architecture of type 2 diabetes susceptibility. *Nat Genet* **46**, 234-44 (2014).
36. Scott, R.A. *et al.* An Expanded Genome-Wide Association Study of Type 2 Diabetes in Europeans. *Diabetes* **66**, 2888-2902 (2017).
37. Bradfield, J.P. *et al.* A genome-wide meta-analysis of six type 1 diabetes cohorts identifies multiple associated loci. *PLoS Genet* **7**, e1002293 (2011).
38. Florez, J.C. Genetics and biobanks converge to resolve a vexing knowledge gap in diabetes. *Lancet Diabetes Endocrinol* **6**, 87-89 (2018).
39. Cervin, C. *et al.* Genetic similarities between latent autoimmune diabetes in adults, type 1 diabetes, and type 2 diabetes. *Diabetes* **57**, 1433-1437 (2008).
40. Saxena, R. *et al.* Large-Scale Gene-Centric Meta-Analysis across 39 Studies Identifies Type 2 Diabetes Loci (vol 90, pg 410, 2012). *American Journal of Human Genetics* **90**, 753-753 (2012).
41. Wu, Y., Zheng, Z., Visscher, P.M. & Yang, J. Quantifying the mapping precision of genome-wide association studies using whole-genome sequencing data. *Genome biology* **18**, 86 (2017).
