# Supplementary material for: Genome-wide association analyses identify 143 risk variants and putative regulatory mechanisms for type 2 diabetes
Source: Nat Commun. 2018 Jul 27;9:2941. doi: 10.1038/s41467-018-04951-w (PMC6063971; doi:10.1038/s41467-018-04951-w)
Supplement: Supplementary file 2 — Description of Additional Supplementary Files [file 41467_2018_4951_MOESM2_ESM.pdf]

Description of Supplementary Data

File Name: Supplementary Data 1

Description: Independent common variants associated with T2D at $P < 5E\text{-}8$ from the meta-analysis.

File Name: Supplementary Data 2

Description: Gene loci identified by gene-based association test using GCTA-fastBAT.

File Name: Supplementary Data 3

Description: Common variants identified by GCTA-COJO analysis of the meta-analysis summary data at $P_{COJO} < 5e\text{-}8$.

File Name: Supplementary Data 4

Description: Multiple regression analysis of SNPs identified from the GCTA-COJO analysis

File Name: Supplementary Data 5

Description: Enrichment of the variance explained by SNPs in different functional categories for T2D.

File Name: Supplementary Data 6

Description: Gene-set enrichment analysis of the meta-analysis data by MAGMA.

File Name: Supplementary Data 7

Description: Enrichment of the T2D-associated genes in protein-protein interaction network.

File Name: Supplementary Data 8

Description: T2D-associated genes identified from the SMR analysis of the GWAS summary data from our meta-analysis and the eQTL summary data from the eQTLGen consortium.

File Name: Supplementary Data 9

Description: T2D-associated genes identified from the SMR analysis of the GWAS summary data from our meta-analysis and the eQTL summary data from the CAGE consortium.

File Name: Supplementary Data 10
Description: T2D-associated CpG methylation sites from the SMR analysis of the GWAS summary data from our meta-analysis and the mQTL data from McRae et al.

File Name: Supplementary Data 11
Description: Mapping the T2D-associated CpG methylation sites to the T2D-associated genes by the SMR analysis of the eQTL data from the eQTLGen consortium and the mQTL data from McRae et al.

File Name: Supplementary Data 12
Description: Mapping the T2D-associated CpG methylation sites to the T2D-associated genes by the SMR analysis of the eQTL data from the CAGE consortium and the mQTL data from McRae et al.

File Name: Supplementary Data 13
Description: Replication of the T2D-associated genes identified using the eQTLGen data in five GTEx tissues.

File Name: Supplementary Data 14
Description: Effects of the lead SNPs at the three putative T2D drug target gene loci on insulin-related and lipids traits.

File Name: Supplementary Data 15
Description: T2D association statistics of the known T1D susceptibility loci.
